# Acute Change of Footwear Limits Performance and Alters Foot Strike Patterns During Anticipated and Unanticipated 90° Change of Direction

**DOI:** 10.1002/jfa2.70103

**Published:** 2025-12-03

**Authors:** Stanislav Dimitri Siegel, Mareike Sproll, Konstantin Warneke, Joel Mason, Astrid Zech

**Affiliations:** ^1^ Department of Human Movement Science and Exercise Physiology Institute of Sport Science Friedrich Schiller University Jena Jena Germany; ^2^ Department of Human Movement Science University of Hamburg Hamburg Germany

**Keywords:** anticipation, barefoot, change of direction, footwear, minimalist shoes

## Abstract

**Background:**

Footwear influences performance and injury risk during change‐of‐direction (COD) movements. However, few studies have investigated how different shoe types influence COD biomechanics, particularly under ecologically valid conditions such as at sharp angles and under unanticipated conditions. This study examined the impact of barefoot, minimalist, and standard sport shoes on biomechanical and performance parameters during 90° COD tasks.

**Methods:**

Forty‐one participants (14 females and 27 males) completed a randomized crossover study, performing anticipated and unanticipated 90° COD tasks under three footwear conditions: barefoot, minimalist shoes, and habitual standard sport shoes. Kinematic, kinetic, and lower leg muscle activity data were collected using motion capture, force plates, and surface electromyography.

**Results:**

Standard sport shoes resulted in faster COD completion times, significantly higher approach velocity, and greater braking and propulsion forces than minimalist and barefoot condition (*p* < 0.05). Minimalist and barefoot condition led to altered foot strike patterns, reduced knee flexion, and lower GRF magnitudes. Unanticipated trials increased neuromuscular demand, reducing approach velocity and altering GRF distribution across all footwear conditions. After controlling for approach velocity, footwear effects remained significant in most kinetic and kinematic measures.

**Conclusion:**

These findings highlight the importance of footwear choice for performance and biomechanical outcomes during challenging COD tasks.

## 1 Introduction

Change‐of‐direction (COD) is a critical movement underpinning success in various sports [1, 2], occurring approximately 50 times per soccer game [1] and 1.6 times per point in tennis [3]. COD is associated with a high incidence of injuries, including anterior cruciate ligament (ACL) tears [4], ankle sprains [5, 6], and muscle strains [7, 8], which frequently occur in noncontact situations within the first 50 milliseconds of foot contact [4]. Therefore, initial foot contact is a critical phase, as high ground reaction forces (GRF) are applied to the braking limb [9]. The distribution of GRF along the lower limb is significantly influenced by the foot strike pattern (FSP). Uno et al. [10] demonstrated that FSP affects how GRF interacts with the body during a 45° COD. In rearfoot strikes (RFS), participants exhibited a more extended knee at landing, whereas forefoot strikes (FFS) involved greater knee flexion, leading to an altered GRF direction than RFS and, consequently, different loading patterns on the knee joint. Other studies further support the link between FSP and changes in kinetic and energetic distribution within the lower extremities [11, 12, 13].

Research in running biomechanics suggests that FSP is influenced by footwear choice [14, 15]. Acute transitions from familiar cushioned shoes to barefoot or minimalist footwear induce alterations in sagittal plane joint kinematics, most consistently characterized by a flatter foot strike pattern during linear running [15, 16, 17]. These adaptations are typically accompanied by greater knee flexion at initial contact, reduced knee range of motion (ROM) [17, 18], and increased ankle ROM [16, 17, 18]. Such kinematic adjustments likely reflect changes in leg stiffness, with FFS associated with increased knee stiffness and a more compliant ankle joint [19, 20]. This adaptation occurs as a compensation strategy for the lack of external cushioning, increasing reliance on the lower leg musculature for shock absorption [20, 21, 22].

Most footwear research in sports has focused on running, with limited studies on game‐related movements such as COD. Sinclair et al. [23, 24] found that minimalist footwear increased impact loading in 45° COD and altered ankle kinematics in 180° CODs compared with court‐specific shoes. Specifically, in 45° CODs, minimalist shoes produced higher average and instantaneous loading rates without substantial differences in joint kinematics, whereas in 180° CODs, participants exhibited a more plantarflexed ankle position, greater ankle ROM, and increased inversion and internal rotation in the minimalist footwear than conventional sport shoes. Stacoff et al. [25] reported better lateral stability of the ankle joint in barefoot condition (less inversion) than three standard court shoes differing in sole properties and two prototype models. The prototype shoes featured specialized rearfoot constructions: one with a noncompressible double inner sole allowing slipping between layers and another with a hollow inner core and a rubber bottom enabling the lateral walls to deform under load. During a 45° COD, barefoot performance showed superior stability, challenging the assumption that specialized footwear necessarily enhances safety.

However, the impact of these findings is limited by small sample sizes (*n* < 13) and the focus on shallower or extreme COD angles, whereas 90° COD movements have not yet been systematically examined. Biomechanically, sharper CODs require greater braking forces and altered knee and ankle kinematics to control deceleration and reorientation [26, 27, 28]. Specifically, 90° CODs generate higher mediolateral GRF and lower knee flexion angles than shallower 45° COD, indicating greater mechanical demands [26, 29]. Additionally, research has predominantly focused on anticipated CODs, whereas unanticipated CODs impose greater demands on neuromuscular control and movement execution [29, 30, 31, 32]. Given that both sharper COD angles and unanticipated movements require distinct biomechanical adaptations, it is essential to examine how different footwear conditions influence these parameters.

To address this research gap, this study aims to investigate the influence of barefoot condition, minimalist footwear, and standard sport shoes on performance and ankle and knee kinematics, kinetics, and lower leg muscle activity at initial ground contact and during the stance phase during both anticipated and unanticipated 90° COD movements. We hypothesize that an acute transition from familiar cushioned footwear to barefoot or minimalist shoe will not only alter biomechanical factors, but also affect performance, leading to prolonged movement execution times.

## 2 Methods

A randomized crossover study was conducted in which all participants completed COD tests in a biomechanics laboratory, following a randomized footwear sequence. Participants were instructed to arrive at the laboratory at least 3 h postprandial, fully hydrated, and to refrain from strenuous exercise for 48 h prior to testing. All measurements and preparations, including marker placement, were performed by the same researchers to ensure consistency.

Active, healthy, and habitually shod females and males were recruited from local sports clubs and universities. Eligibility criteria required participants to be regularly engaged in a nonbarefoot sport at the time of the study. Exclusion criteria included any lower extremity injury within the past 6 months, clinically diagnosed foot deformities, motor‐functional impairments, or habitual use of minimalist footwear prior to the study.

Ethical approval was obtained from the local university ethics committee (protocol number FSV 22/066), and all procedures adhered to the principles outlined in the Declaration of Helsinki.

### 2.1 Footwear Conditions

**FIGURE 1:**
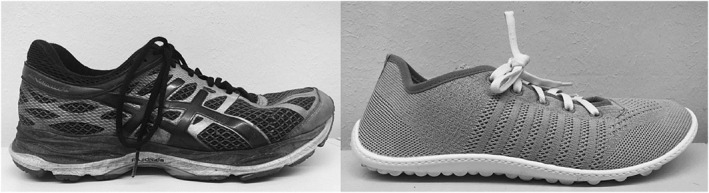
Example of a traditional running shoe (left) versus a minimalist shoe (right).

The footwear conditions were classified into three categories: barefoot, minimalist shoes (leguano GO, leguano GmbH, Germany), and standard sport shoes (Figure 1). For the standard sport shoe condition, participants wore their own habitual cushioned sport shoes (Supporting Information S1: Table 1, Appendix).

### 2.2 Procedure

The warm‐up and the familiarization phase consisted of 20 trials of the 90° COD test, performed with progressive increases in intensity, followed by three countermovement jumps and three single‐leg drop jumps per leg. All tests were explained and demonstrated by the same examiner, who provided feedback for incorrect trials. A minimum rest period of 1 min was maintained among trials and 5 min between footwear conditions. To minimize potential fatigue effects, the sequence of tests and the starting leg were randomized.

#### 2.2.1 90° Cutting Maneuver (90° COD)

**FIGURE 2:**
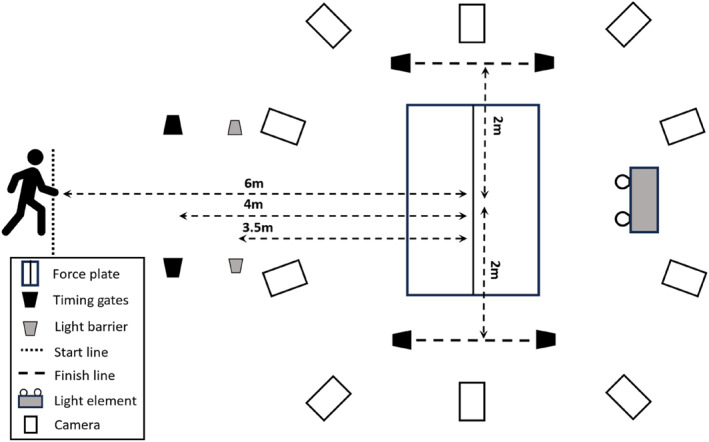
Experimental setup for 90° cutting maneuver (90° COD).

To evaluate the anticipated 90° COD, photoelectric sensors were positioned 4 m in front of the force plate's center, with an additional setup 2 m to each side (Figure 2). Participants initiated their movement from a starting point 6 m ahead of the force plate's center, allowing a 2 m approach before passing through the first set of sensors.

To perform a valid 90° COD, the last foot contact (plant step) had to occur within a designated 70 × 100 cm zone on the force plate. For a leftward COD, this contact was made with the right foot, followed by an immediate 90° pivot to the left and a quick movement through the second set of photoelectric sensors, positioned 2 m away. For a rightward COD, contact was made with the left foot. Flexibility in the exact starting point was permitted to account for individual differences in stride patterns during approach. Participants were instructed to approach the force platform on a straight path.

The unanticipated 90° COD task was conducted similarly to the anticipated 90° COD, with the addition of a customized knee‐height light barrier 3.5 m before the force plate. Breaking the beam triggered a random left/right response instruction, which was positioned 2 m behind the force plate at eye level. Pilot testing optimized barrier placement for maximum approach velocity and sufficient reaction time. Participants were instructed in both conditions to run through the second pair of timing gates as fast as possible. In total, participants completed four valid trials for each of the six conditions (3 footwear × 2 anticipation types), resulting in 24 trials per participant.

### 2.3 Instruments

#### 2.3.1 Surface Electromyography (sEMG)

A wireless electromyography system (Ultium EMG, Noraxon, USA) was used to record muscle activity for the tibialis anterior (TA), lateral gastrocnemius (GL), and medial gastrocnemius (GM) muscles. Data were sampled at 2000 Hz, and the skin was shaved and cleaned. A skin prepping gel was applied, and disposable Ag/AgCl surface electrodes (Dual‐Electrodes, Noraxon, USA) placed 20 mm apart were placed on standardized locations bilaterally on measured muscles according to the surface EMG guidelines [33, 34]. To prevent slippage during movement, three layers of elastic cohesive bandage (Peha‐haft, Germany) and one layer of kinesiotape (Leukotape, Germany) were applied over the electrodes and sensors. In addition, Velcro elasticated wraps were attached to the lower edge of the surface EMG (sEMG) sensors. An impendency measurement was taken to ensure a value below 10 kΩ. The baseline noise level was visually checked before each trial.

#### 2.3.2 Motion Capture

Participants were measured wearing tight‐fitting shorts and shirtless or with a crop top. Reflective markers (12 and 5 mm) were placed on the following landmarks: iliac crest, anterior superior iliac spine; posterior superior iliac spine; medial epicondyle and lateral epicondyle of the femur; lateral malleolus, medial malleolus; posterior calcaneus; and fifth, second and first metatarsal heads using double‐sided adhesive tape. On the shoes, markers were attached to the fabric at the first, second, and fifth metatarsal heads and the superior part of the posterior calcaneus.

An additional set of 4 marker clusters (4 retroreflective markers attached to a lightweight rigid plastic shell) were placed on the right and the left thigh and the shank using velcro elasticated wraps to approximate the movement of these segments during dynamic testing [35, 36, 37]. For the dynamic measurements, the markers on the medial malleolus and medial epicondyle were removed.

To minimize potential bias caused by heel‐to‐toe offsets between footwear conditions, the heel and forefoot markers were carefully positioned on a consistent horizontal plane across all shoes.

Three‐dimensional marker position data were collected using 10 infrared cameras (type Oqus 700) at 300 Hz; GRF data were collected from two force platforms (1.70 × 0.5 m) embedded into the instrumented treadmill (Bertec, USA) sampling at 2700 Hz. The force plates’ surface was level with the approach runway. All measurement systems were acquired and synchronized by Qualisys Track Manager software (Qualisys AB, Version 2019.2, Sweden). A wireless infrared timing system (TC, Brower Timing Systems, USA) consisting of three photoelectric sensors, and TCi Timer was used to record the time to complete the 90° COD. The photoelectric sensors were mounted on tripods 110 cm above the ground and positioned as shown in Figure 2.

### 2.4 Data Analysis

Visual3D software (C‐motion, v2021.03.2) was used to process the force plate and marker data. The force plate and marker data were low‐pass filtered using a fourth‐order Butterworth filter at 25 and 12 Hz, respectively [38]. Force data were normalized to the body weight.

A 6‐degrees‐of‐freedom kinematic model of the lower extremity was created from a static trial, encompassing pelvis, thigh, shank, and foot using cone geometry. The pelvis was modeled as a cylinder, and the CODA pelvis orientation [39] served to estimate hip joint center location's via standard regression equations. The knee and ankle joint centers were defined as the midpoint of the line connecting the lateral and medial markers. This kinematic model was designed to quantify the motion of the hip, knee, and ankle joints using a cardan angle sequence in the order of *x*‐*y*‐*z* [40]. The local coordinate system was defined at the proximal joint center. The global coordinate system was defined according to the approach direction as follows: anterior–posterior = *Y*‐direction, mediolateral = *X*‐direction, superior–inferior = *Z*‐direction. The static trial position was designated as the subject's neutral alignment, with subsequent joint angles normalized to this position.

Processed model data were exported to MATLAB R2023a (The MathWorks, USA) for further analysis. The sEMG signals were processed using a band‐pass filter with cutoff frequencies set at 10 Hz for the high‐pass filter and 500 Hz for the low‐pass filter. Following filtration, the signals were rectified to obtain the positive half‐wave. The root mean square values were computed using a 50‐ms moving window [34].

Ground contact and toe‐off events were identified by analyzing force data in conjunction with marker data. Ground contact was defined as the time point at which the vertical GRF exceeded 50 N, whereas toe‐off was identified by a subsequent drop below 50°N [41]. The braking phase was defined as the period from initial ground contact to the time point at which the knee flexion angle reached its maximum during stance, whereas the subsequent period until the toe‐off phase was defined as the propulsive phase [42]. The mean normalized center‐of‐pressure (COP) position during the first 5% of ground contact time was calculated to classify the FSP. A mean COP value between 0 and 0.33 indicated a rearfoot strike RFS, between 0.34 and 0.66 indicated an MFS, and above 0.66 indicated an FFS [43]. The COP data were normalized by dividing the *x* and *y* coordinates by foot width and length, respectively. The transformation of the COP data into the foot coordinate system with the coordinate origin at the calcaneal marker was performed using the foot rotation matrix extracted at 20% of ground contact time, ensuring ground contact of the whole foot. A virtual marker for the center of the sacrum was calculated as the midpoint between right and left posterior–superior iliac spine markers and used as a pelvis reference to compute whole‐body horizontal velocity (approach/toe‐off) and the COD angle across trials. In Supporting Information S1, Table 2 presents the definition of all dependent variables. The calculated variables were exported to a Microsoft Excel (Microsoft Corporation, USA) file for further statistical analysis.

### 2.5 Statistics

Statistical analysis was conducted using RStudio, version 4.0.3 (RStudio, Boston). Participants with less than two valid trials for each shoe condition, direction, and anticipation condition were excluded. For each dependent variable, a linear mixed‐effect model (LMM) was fitted using the lmer function from the lme4 package [44]. Model assumptions (normality and homoscedasticity) were visually assessed via q‐q and residual scatterplots [45]. A robust linear mixed model confirmed result stability, and LMM was retained as the primary model.

The main model included shoe and anticipation conditions as fixed effects, with their interaction, and a random intercept for each participant. Since approach velocity was not controlled, an additional model included it as a covariate to determine whether shoe or anticipation effects remained significant. Model fit was evaluated via predicted versus actual plots, and marginal/conditional *R* ^2^ values estimated explanatory power (Supporting Information S1: Table 3). An analysis of variance (ANOVA) was performed to assess the fixed effects for both models, and significant findings were followed by Bonferroni‐adjusted pairwise comparisons. A *p* value < 0.05 was considered statistically significant. The effect sizes were calculated using eta square (*η* ^2^): 0.01 for small, 0.06 for medium, and 0.14 for large [46].

## 3 Results

Forty‐eight participants initially volunteered, seven of whom were excluded for insufficient data in at least one condition. Table 1 presents the sample characteristics of all included participants. A total of 1859 movement trials were analyzed, including 617 with minimalist shoes, 612 barefoot, and 630 with standard sport shoes.

**TABLE 1:** Demographic and physical characteristics of participants (mean ± standard deviation).

		Females	Males	All
		*n* = 14	*n* = 27	*n* = 41
Age [y]		20.68 ± 2.51	22.31 ± 4.85	21.75 ± 4.27
Height [cm]		168.34 ± 4.36	181.42 ± 7.83	176.95 ± 9.24
Body weight [kg]		62.27 ± 8.52	79.51 ± 12	73.62 ± 13.66
BMI [kg*m^−2^]		21.94 ± 2.62	24.07 ± 2.82	23.34 ± 2.93
Years of sport activity [y]		9.44 ± 5.33	14.09 ± 5.35	12.50 ± 5.78
Leg dominance	Left	0	7	7
	Right	14	20	34

Abbreviation: BMI, body mass index.

### 3.1 Spatiotemporal Characteristics and Performance

**FIGURE 3:**
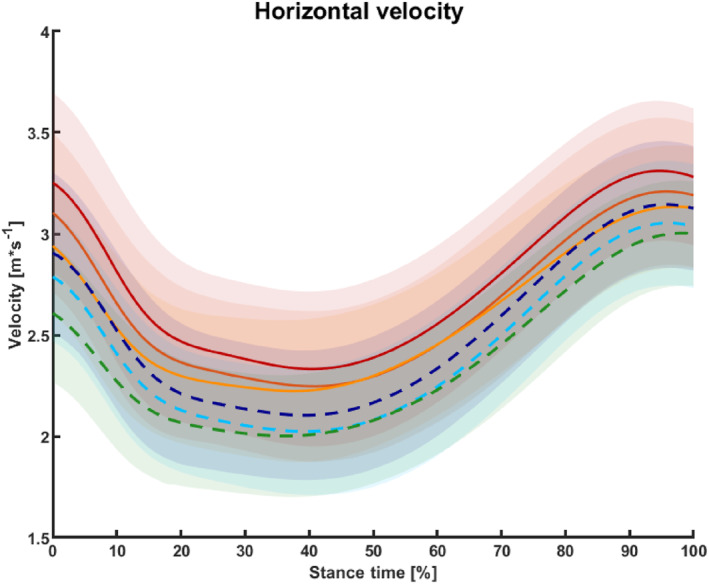
Interpolated mean and standard deviation of velocity curves during the stance phase of the 90° change‐of‐direction task across footwear and anticipation conditions. Warm‐colored solid lines represent the anticipated condition, and cool‐colored dashed lines represent the unanticipated condition. The standard sport shoe condition is shown in red and dark blue, the minimalist shoe condition in orange‐red and cyan, and the barefoot condition in orange and green.

**FIGURE 4:**
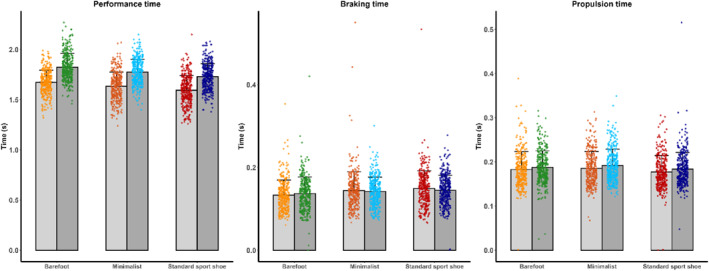
Mean and standard deviation of performance time (left), braking time (middle), and propulsion time (right), presented as scatterplots combined with bar plots. Warm colors represent the anticipated condition, and cool colors represent the unanticipated condition. Point clouds correspond to individual trials in their respective colors. Mean and standard deviation are illustrated by bars, with gray bars indicating the anticipated and dark gray bars indicating the unanticipated condition. Color coding is as follows: standard sport shoe—red (anticipated) and dark blue (unanticipated), minimalist shoe—orange (anticipated) and cyan (unanticipated), and barefoot—yellow (anticipated) and green (unanticipated).

Participants achieved significantly higher approach velocity in standard sport shoes than minimalist and barefoot condition (*p* < 0.001) (Figure 3). Approach velocity was lower under unanticipated conditions across all footwear types (Figure 4). Both footwear and anticipation significantly affected performance time (*p* < 0.001), with standard sport shoes yielding the fastest completion times in both anticipation states. Controlling for approach velocity reduced the effect size but did not eliminate the advantage of standard shoes.

Standard shoes also produced significantly greater toe‐off velocity than minimalist or barefoot footwear (*p* < 0.001). An anticipation effect persisted even after controlling for approach velocity.

The COD angle was greatest in standard shoes and smallest in barefoot trials (*p* < 0.001). Although the anticipation effect was significant in the unadjusted model, it became nonsignificant after controlling for approach velocity.

Although absolute differences in GCT across footwear were minimal, they reached statistical significance (*p* < 0.05), with barefoot COD showing slightly shorter GCT. Anticipation had a significant effect on the model without the approach velocity covariate but not after adjustment. Barefoot COD demonstrated shorter braking times, whereas minimalist footwear showed longer propulsion times (*p* < 0.001). These effects remained significant with approach velocity as a covariate. A significant interaction (*p* < 0.05) showed that footwear differences were prominent in anticipated trials but diminished or disappeared in unanticipated trials (Table 2).

**TABLE 2:** ANOVA results for spatiotemporal parameters—effects of footwear and anticipation with and without approach velocity as a covariate.

		Anticipation		ANOVA without covariate				ANOVA with covariate			
		With	Without	F (df1, df2)	*p* value	Conditional/marginal *R* ^2^	*η* ^2^	F (df1, df2)	*p* value	Conditional/marginal *R* ^2^	*η* ^*2*^
Performance time [s]											
Footwear	Standard	1.59 ± 0.15	1.74 ± 0.13	107.9 (2,1814.5)	*p* < 0.001^a^ ^,^ ^b^ ^,^ ^c^	0.580/0.257	0.11	30.1 (2,1824.7)	*p* < 0.001^a^ ^,^ ^b^ ^,^ ^c^	0.599/0.395	0.03
	Minimalist	1.63 ± 0.14	1.78 ± 0.13								
	Barefoot	1.67 ± 0.12	1.83 ± 0.14								
Anticipation				923.9 (1,1813.3)	*p* < 0.001		0.34	339.4 (1,1843.8)	*p* < 0.001		0.16
Approach velocity								273.2 (1,1813.0)	*p* < 0.001		0.13
Approach velocity [m*s^−1^]											
Footwear	Standard	3.28 ± 0.45	2.92 ± 0.39	179.3 (2,1814)	*p* < 0.001^a^ ^,^ ^b^ ^,^ ^c^	0.625/0.227	0.17	179.26 (2,1814.0)	*p* < 0.001^a^ ^,^ ^b^ ^,^ ^c^	0.625/0.227	0.17
	Minimalist	3.15 ± 0.40	2.81 ± 0.32								
	Barefoot	2.99 ± 0.34	2.64 ± 0.34								
Anticipation				769.3 (1,1813.1)	*p* < 0.001		0.3	768.86 (1,1813.1)	*p* < 0.001		0.3
Approach velocity											
Toe‐off velocity [m*s^−1^]											
Footwear	Standard	3.29 ± 0.33	3.12 ± 0.30	49.65 (2,1815.2)	*p* < 0.001^a^ ^,^ ^b^ ^,^ ^c^	0.427/0.084	0.05	9.50 (2,1825.0)	*p* < 0.001^a^ ^,^ ^b^	0.475/0.202	0.01
	Minimalist	3.20 ± 0.36	3.04 ± 0.31								
	Barefoot	3.13 ± 0.30	3.00 ± 0.26								
Anticipation				174.90 (1,1813.7)	*p* < 0.001		0.09	15.64 (1,1843.7)	*p* < 0.001		0.008
Approach velocity								209.85 (1,1816.4)	*p* < 0.001		0.1
Change‐of‐direction angle [°]											
Footwear	Standard	54.63 ± 6.09	55.56 ± 7.67	52.86 (2,1815.3)	*p* < 0.001^a^ ^,^ ^b^ ^,^ ^c^	0.351/0.047	0.06	100.50 (2,1826.1)	*p* < 0.001^a^ ^,^ ^b^ ^,^ ^c^	0.397/0.125	0.1
	Minimalist	52.98 ± 6.02	54.31 ± 6.07								
	Barefoot	50.78 ± 6.20	52.47 ± 7.54								
Anticipation				25.23 (1,1813.3)	*p* < 0.001		0.01	2.62 (1,1846.0)	0.106		0.001
Approach velocity								124.79 (1,1793.6)	*p* < 0.001		0.07
Ground contact time [s]											
Footwear	Standard	0.33 ± 0.07	0.33 ± 0.06	3.50 (2,1814.0)	*p* = 0.030^c^	0.576/0.004	0.004	5.70 (2,1817.9)	0.003^c^	0.573/0.008	0.006
	Minimalist	0.33 ± 0.07	0.34 ± 0.06								
	Barefoot	0.32 ± 0.07	0.33 ± 0.06								
Anticipation				7.69 (1,1813.3)	*p* = 0.006		0.004	0.52 (1,1828.1)	0.471		0.0003
Approach velocity								8.85 (1,1850.5)	0.003		0.005
Braking time [s]^d^											
Footwear	Standard	0.15 ± 0.04	0.15 ± 0.04	18.93 (2,1815.0)	*p* < 0.001^a^ ^,^ ^b^ ^,^ ^c^	0.399/0.014	0.02	19.93 (2,1823.1)	*p* < 0.001^a^ ^,^ ^b^ ^,^ ^c^	0.394/0.016	0.02
	Minimalist	0.14 ± 0.05	0.14 ± 0.04								
	Barefoot	0.13 ± 0.04	0.14 ± 0.04								
Anticipation				0.07 (1,1813.5)	*p* = 0.792		< 0.001	1.28 (1,1840.3)	0.259		< 0.001
Approach velocity								2.83 (1,1834.9)	0.093		0.002
Propulsion time (s)											
Footwear	Standard	0.18 ± 0.03	0.18 ± 0.04	16.46 (2,1814.5)	*p* < 0.001^a^ ^,^ ^b^	0.472/0.015	0.02	10.39 (2,1820.7)	*p* < 0.001^a^ ^,^ ^b^	0.472/0.020	0.01
	Minimalist	0.19 ± 0.04	0.19 ± 0.04								
	Barefoot	0.18 ± 0.04	0.19 ± 0.04								
Anticipation				20.57 (1,1813.4)	*p* < 0.001		0.01	5.20 (1,1834.7)	0.023		0.003
Approach velocity								8.12 (1,1850.0)	0.004		0.004

^a^ Post hoc test significantly different (*p* < 0.05) between standard sport shoes and minimalist shoes.

^b^ Post hoc test significantly different (*p* < 0.05) between standard sport shoes and barefoot.

^c^ Post hoc test significantly different (*p* < 0.05) between minimalist shoes and barefoot.

^d^ ANOVA significant interaction effect (*p* < 0.05) between anticipation and footwear.

#### 3.1.1 Kinetic Variables

Footwear significantly influenced kinetic variables (Table 3), with most showing moderate to high effect sizes. Standard sport shoes consistently produced higher GRF than minimalist or barefoot condition across mean, maximum braking, and maximum propulsion measures (*p* < 0.001). Although approach velocity explained much of the variance, particularly in forward and vertical force components, footwear effects remained unexplained except for maximum propulsion force in the vertical direction.

**TABLE 3:** ANOVA results for kinetic parameters—effects of footwear and anticipation with and without approach velocity as a covariate.

		Anticipation		ANOVA without covariate				ANOVA with covariate			
		With	Without	F (df1, df2)	*p* value	Conditional/marginal *R* ^2^	*η* ^2^	F (df1, df2)	*p* value	Conditional/marginal *R* ^2^	*η* ^2^
Dynamic friction coefficient											
Footwear	Standard	0.57 ± 0.06	0.58 ± 0.06	489.04 (2,1814.9)	*p* < 0.001^a^ ^,^ ^b^ ^,^ ^c^	0.523/0.257	0.35	313.02 (2,1826.2)	*p* < 0.001^a^ ^,^ ^b^ ^,^ ^c^	0.535/0.333	0.26
	Minimalist	0.55 ± 0.05	0.55 ± 0.04								
	Barefoot	0.50 ± 0.04	0.51 ± 0.05								
Anticipation				2.39 (1,1813.2)	*p* = 0.123		0.001	58.53 (1,1846.6)	*p* < 0.001		0.03
Approach velocity								138.23 (1,1785.6)	*p* < 0.001		0.07
Mean force in sideward direction [BW]											
Footwear	Standard	−0.55 ± 0.09	−0.59 ± 0.10	231.88 (2,1815.1)	*p* < 0.001^a^ ^,^ ^b^ ^,^ ^c^	0.473/0.172	0.20	174.13 (2,1824.0)	*p* < 0.001^a^ ^,^ ^b^ ^,^ ^c^	0.473/0.176	0.16
	Minimalist	−0.52 ± 0.09	−0.55 ± 0.08								
	Barefoot	−0.47 ± 0.08	−0.51 ± 0.08								
Anticipation				126.64 (1,1813.4)	*p* < 0.001		0.07	121.09 (1,1842.1)	*p* < 0.001		0.06
Approach velocity								7.81 (1,1825.6)	0.005		0.004
Mean force in forward direction [BW]											
Footwear	Standard	−0.50 ± 0.10	−0.46 ± 0.09	311.79 (2,1815.2)	*p* < 0.001^a^ ^,^ ^b^ ^,^ ^c^	0.510/0.219	0.26	157.26 (2,1827.5)	*p* < 0.001^a^ ^,^ ^b^ ^,^ ^c^	0.550/0.370	0.15
	Minimalist	−0.47 ± 0.08	−0.42 ± 0.08								
	Barefoot	−0.41 ± 0.07	−0.37 ± 0.07								
Anticipation				202.95 (1,1813.7)	*p* < 0.001		0.10	13.45 (1,1848.0)	*p* < 0.001		0.007
Approach velocity								296.91 (1,1768.5)	*p* < 0.001		0.14
Mean force in vertical direction [BW]											
Footwear	Standard	1.39 ± 0.13	1.39 ± 0.14	31.56 (2,1814.6)	*p* < 0.001^b^ ^,^ ^c^	0.479/0.019	0.03	12.64 (2,1820.5)	*p* < 0.001^b^ ^,^ ^c^	0.490/0.039	0.01
	Minimalist	1.38 ± 0.13	1.38 ± 0.13								
	Barefoot	1.36 ± 0.12	1.35 ± 0.14								
Anticipation				2.55 (1,1813.5)	*p* = 0.11		0.001	3.75 (1,1834.2)	0.053		0.002
Approach velocity								37.10 (1,1850.6)	*p* < 0.001		0.02
Maximum braking force in sideward direction [BW]											
Footwear	Standard	−0.79 ± 0.18	−0.85 ± 0.21	363.71 (2,1814.7)	*p* < 0.001^a^ ^,^ ^b^ ^,^ ^c^	0.527/0.203	0.29	250.37 (2,1822.6)	*p* < 0.001^a^ ^,^ ^b^ ^,^ ^c^	0.535/0.229	0.22
	Minimalist	−0.68 ± 0.15	−0.72 ± 0.15								
	Barefoot	−0.61 ± 0.13	−0.65 ± 0.14								
Anticipation				54.74 (1,1813.3)	*p* < 0.001		0.03	101.78 (1,1839.2)	*p* < 0.001		0.05
Approach velocity								49.13 (1,1839.1)	*p* < 0.001		0.03
Maximum braking force in forward direction [BW]^d^											
Footwear	Standard	−1.17 ± 0.34	−0.99 ± 0.30	302.61 (2,1815.3)	*p* < 0.001^a^ ^,^ ^b^ ^,^ ^c^	0.479/0.221	0.25	129.48 (2,1828.8)	*p* < 0.001^a^ ^,^ ^b^ ^,^ ^c^	0.612/0.476	0.12
	Minimalist	−1.01 ± 0.24	−0.88 ± 0.23								
	Barefoot	−0.83 ± 0.17	−0.75 ± 0.18								
Anticipation				171.11 (1,1813.4)	*p* < 0.001		0.09	0.53 (1,1849.7)	0.465		< 0.001
Approach velocity								638.55 (1,1733.5)	*p* < 0.001		0.27
Maximum braking force in vertical direction [BW]											
Footwear	Standard	2.36 ± 0.48	2.29 ± 0.47	91.88 (2,1815.3)	*p* < 0.001^a^ ^,^ ^b^ ^,^ ^c^	0.389/0.068	0.09	22.63 (2,1825.1)	*p* < 0.001^b^ ^,^ ^c^	0.488/0.230	0.02
	Minimalist	2.29 ± 0.42	2.23 ± 0.40								
	Barefoot	2.11 ± 0.36	2.03 ± 0.37								
Anticipation				20.40 (1,1813.5)	*p* < 0.001		0.01	28.16 (1,1844.1)	*p* < 0.001		0.02
Approach velocity								306.78 (1,1812.2)	*p* < 0.001		0.14
Maximum propulsion force in sideward direction [BW]											
Footwear	Standard	−0.83 ± 0.13	−0.87 ± 0.14	120.17 (2,1815.2)	*p* < 0.001^a^ ^,^ ^b^ ^,^ ^c^	0.417/0.096	0.12	90.75 (2,1824.3)	*p* < 0.001^a^ ^,^ ^b^ ^,^ ^c^	0.418/0.098	0.09
	Minimalist	−0.79 ± 0.12	−0.82 ± 0.11								
	Barefoot	−0.74 ± 0.12	−0.78 ± 0.13								
Anticipation				57.06 (1,1813.5)	*p* < 0.001		0.03	54.48 (1,1842.5)	*p* < 0.001		0.03
Approach velocity								3.52 (1,1823.2)	*p* = 0.061		0.002
Maximum propulsion force in forward direction [BW]											
Footwear	Standard	−0.68 ± 0.21	−0.63 ± 0.17	67.80 (2,1814.8)	*p* < 0.001^a^ ^,^ ^b^ ^,^ ^c^	0.462/0.072	0.07	22.03 (2,1823.7)	*p* < 0.001^b^ ^,^ ^c^	0.460/0.146	0.02
	Minimalist	−0.66 ± 0.16	−0.59 ± 0.15								
	Barefoot	−0.61 ± 0.14	−0.54 ± 0.15								
Anticipation				110.38 (1,1813.5)	*p* < 0.001		0.06	9.99 (1,1851.5)	0.002		0.005
Approach velocity								122.28 (1,1829.0)	*p* < 0.001		0.06
Maximum propulsion force in vertical direction [BW]											
Footwear	Standard	1.79 ± 0.26	1.80 ± 0.26	3.70 (2,1814.3)	*p* = 0.025^b^	0.510/0.003	0.004	2.42 (2,1819.5)	0.089	0.509/0.003	0.003
	Minimalist	1.79 ± 0.25	1.78 ± 0.24								
	Barefoot	1.79 ± 0.24	1.77 ± 0.26								
Anticipation				0.21 (1,1813.4)	*p* = 0.647		0.0001	0.0007 (1,1831.7)	0.979		< 0.001
Approach velocity								0.576 (1,1852.0)	0.448		< 0.001
Vertical instantaneous load [BW*s^−1^]^d^											
Footwear	Standard	147.2 ± 65.33	146.9 ± 65.69	209.49 (2,1813.6)	*p* < 0.001^a^ ^,^ ^b^ ^,^ ^c^ ^,^ ^d^	0.398/0.1444	0.19	260.74 (2,1826.5)	*p* < 0.001^a^ ^,^ ^b^ ^,^ ^d^	0.424/0.197	0.22
	Minimalist	227.65 ± 86.61	210.61 ± 96.38								
	Barefoot	224.82 ± 87.26	201.87 ± 86.52								
Anticipation				18.62 (1,1812.4)	*p* < 0.001		0.0112	1.66 (1,1847.1)	0.20		0.001
Approach velocity								87.77 (1,1764.2)	*p* < 0.001		0.05
Vertical average load [BW*s^−1^]											
Footwear	Standard	60.63 ± 30.36	53.22 ± 27.24	67.6 (2,1813.5)	*p* < 0.001^a^ ^,^ ^b^ ^,^ ^c^	0.4/0.052	0.07	26.26 (2,1820.3)	*p* < 0.001^a^ ^,^ ^b^	0.492/0.101	0.03
	Minimalist	51.16 ± 33.45	43.76 ± 29.55								
	Barefoot	47.02 ± 34.87	39.14 ± 26.59								
Anticipation				48.57 (1,1812.3)	*p* < 0.001		0.03	0.83 (1,1835.5)	0.360		0.0004
Approach velocity								88.89 (1,1846.0)	*p* < 0.001		0.05

^a^ Post hoc test significantly different (*p* < 0.05) between standard sport shoes and minimalist shoes.

^b^ Post hoc test significantly different (*p* < 0.05) between standard sport shoes and barefoot.

^c^ Post hoc test significantly different (*p* < 0.05) between minimalist shoes and barefoot.

^d^ ANOVA significant interaction effect (*p* < 0.05) between anticipation and footwear.

**FIGURE 5:**
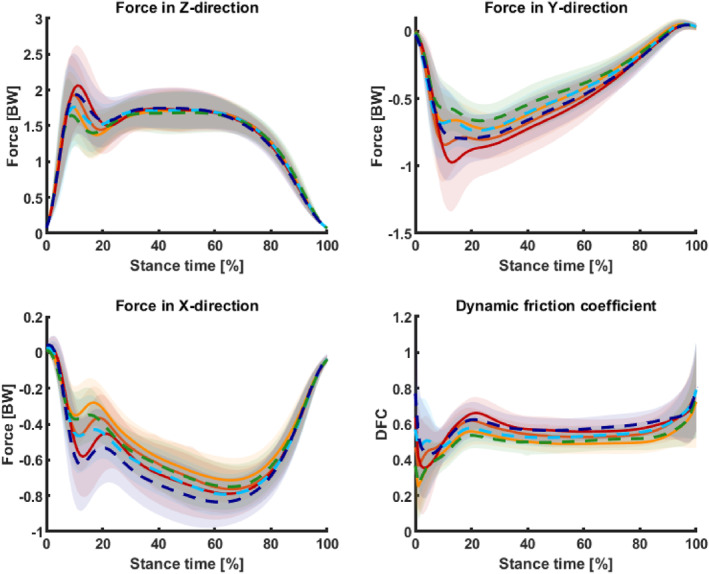
Interpolated mean and standard deviation curves of ground reaction forces in the vertical (*Z*), anterior–posterior (*X*), and medial–lateral (*Y*) directions, as well as the mean dynamic friction coefficient (DFC), during the stance phase of the 90° change‐of‐direction task. Warm‐colored solid lines represent the anticipated condition, whereas cool‐colored dashed lines represent the unanticipated condition. The standard sport shoe condition is shown in red and dark blue, the minimalist shoe condition in orange‐red and cyan, and the barefoot condition in orange and green.

Anticipation lowered mean GRF in the forward direction, as well as maximum braking and propulsion GRF in forward and vertical directions. However, mean and maximum braking and propulsion GRF in the sideward direction were higher under unanticipated conditions (*p* < 0.001). The anticipation effect on maximum braking force in the forward direction diminished or lost significance when controlling for approach velocity (Figure 5).

**FIGURE 6:**
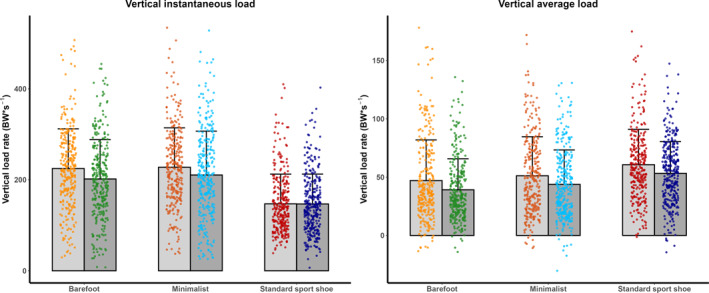
Mean and standard deviation of vertical instantaneous load (left) and vertical average load (right) displayed as scatterplots combined with bar plots. Warm colors represent the anticipated condition, and cool colors represent the unanticipated condition. The point clouds correspond to individual trials in their respective colors. Mean and standard deviation are indicated by bars, with gray bars representing the anticipated and dark gray bars representing the unanticipated condition. Color coding is as follows: standard sport shoe—red (anticipated) and dark blue (unanticipated), minimalist shoe—orange (anticipated) and cyan (unanticipated), and barefoot—yellow (anticipated) and green (unanticipated).

Instantaneous loading was lower in standard sport shoes than in minimalist or barefoot condition (*p* < 0.001), though vertical average loads were reduced in minimalist and barefoot condition (Figure 6). Both parameters were lower under unanticipated conditions. A significant interaction effect between footwear and anticipation (*p* < 0.05) revealed lower vertical instantaneous load in the barefoot and minimalist shoe conditions in the unanticipated than the anticipated condition, with no difference between these conditions. In contrast, standard sport shoes showed no difference among anticipation conditions and consistently exhibited lower vertical instantaneous loads. When approach velocity was included as a covariate, footwear differences were attenuated but not eliminated, whereas differences among anticipation conditions were insignificant.

The dynamic friction coefficient (DFC) was significantly higher in standard sport shoes than barefoot and minimalist shoe conditions, with no initial anticipation effect. After controlling for approach velocity, footwear differences remained significant, and anticipation effects became significant.

#### 3.1.2 Foot Strike Pattern, Ankle and Knee Joint Angle

**FIGURE 7:**
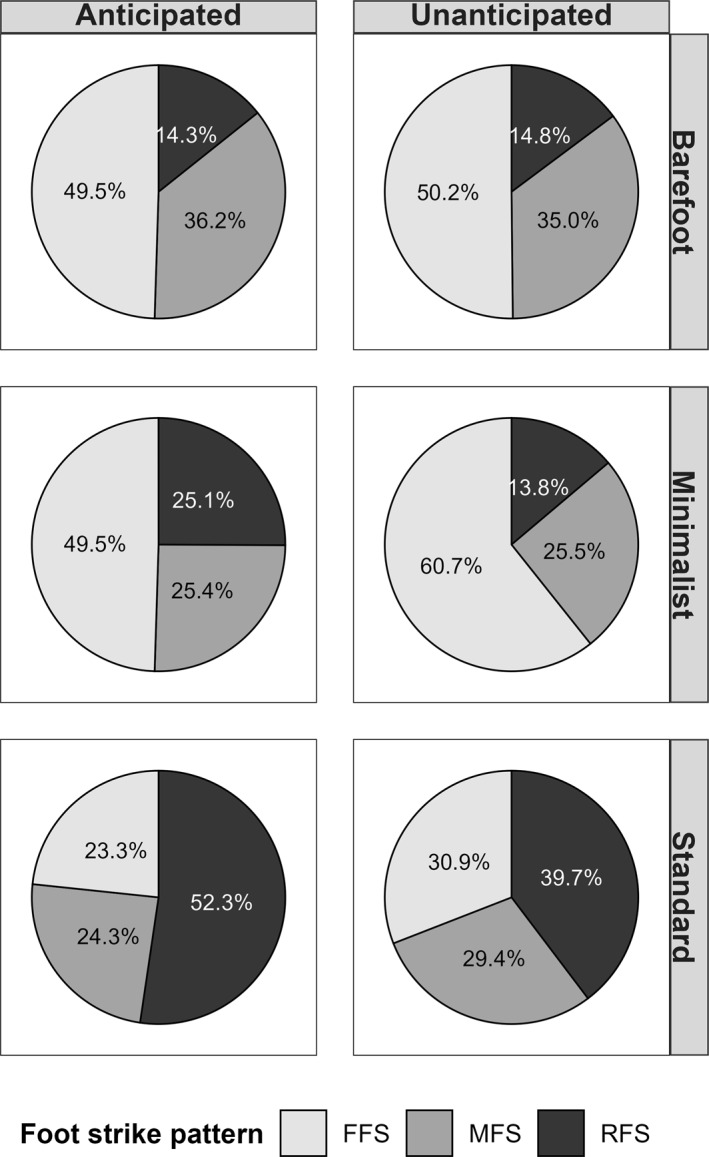
Distribution of foot strike patterns separated by footwear and anticipation conditions.

In the standard footwear condition, rearfoot strikes (RFS) occurred most frequently (≈52% anticipated; 40% unanticipated), whereas minimalist and barefoot condition were predominantly forefoot strikes (FFS > 50%), especially under unanticipated conditions. Midfoot strikes (MFS) generally made up 25%–35% of trials across all conditions but varied slightly by anticipation (Figure 7).

A significant footwear effect with a high effect size was observed for the ankle joint angle, with standard sport shoes showing a more dorsiflexed ankle at initial ground contact than minimalist or barefoot condition (*p* < 0.001). The significant interaction effect (*p* < 0.05) indicated that minimalist and barefoot condition did not differ from each other (regardless of anticipation), yet both were significantly different from the standard sport shoe in both anticipated and unanticipated trials. Anticipation was significant in the unadjusted model (*p* < 0.001) but became nonsignificant after controlling for approach velocity (*p* = 0.132). Ankle ROM differences were small but significant (*p* = 0.002), with slightly greater ROM in standard footwear than barefoot, whereas anticipation and approach velocity had no significant effects (Table 4).

**TABLE 4:** Initial ground contact angle and range of motion during the stance phase of the ankle and knee joint.

		Anticipation		ANOVA without covariate				ANOVA with covariate			
		With	Without	F (df1, df2)	*p* value	Conditional/marginal *R* ^2^	*η* ^*2*^	F (df1, df2)	*p* value	Conditional/marginal *R* ^2^	*η* ^*2*^
Ankle joint angle [°]^d^											
Footwear	Standard	−10.53 ± 9.48	−10.84 ± 9.32	212.70 (2,1814.7)	*p* < 0.001^a^ ^,^ ^b^	0.513/0.117	0.19	207.39 (2,1821.0)	*p* < 0.001^a^ ^,^ ^b^	0.518/0.119	0.19
	Minimalist	−17.96 ± 9.29	−16.71 ± 8.33								
	Barefoot	−18.28 ± 7.62	−16.46 ± 8.42								
Anticipation				11.97 (1,1813.5)	*p* < 0.001		0.007	2.27 (1,1835.3)	0.132		0.001
Approach velocity								6.81 (1,1849.1)	0.009		0.004
Ankle joint range of motion [°]											
Footwear	Standard	44.17 ± 6.32	44.35 ± 6.75	6.27 (2,1814.4)	*p* = 0.002^b^ ^,^ ^c^	0.499/0.005	0.007	6.07 (2,1819.8)	*p* = 0.002^b^ ^,^ ^c^	0.498/0.006	0.007
	Minimalist	43.92 ± 6.35	44.72 ± 6.19								
	Barefoot	43.47 ± 6.73	43.45 ± 6.99								
Anticipation				2.99 (1,1813.5)	*p* = 0.084		0.002	1.35 (1,1832.5)	*p* = 0.245		< 0.001
Approach velocity								0.29 (1,1851.8)	*p* = 0.592		< 0.001
Knee joint angle [°]^d^											
Footwear	Standard	−22.51 ± 6.78	−25.70 ± 8.42	7.23 (2,1815.4)	*p* < 0.001^a^ ^,^ ^b^	0.383/0.051	0.008	2.00 (2,1824.3)	*p* = 0.136	0.421/0.100	0.002
	Minimalist	−23.16 ± 6.33	−26.91 ± 7.15								
	Barefoot	−24.05 ± 6.13	−26.20 ± 7.02								
Anticipation				132.60 (1,1813.7)	*p* < 0.001		0.07	25.88 (1,1842.4)	< 0.001		0.01
Approach velocity								80.43 (1,1824.4)	< 0.001		0.04
Knee joint range of motion [°]											
Footwear	Standard	43.16 ± 8.85	43.46 ± 8.92	20.24 (2,1814.0)	*p* < 0.001^b^ ^,^ ^c^	0.562/0.014	0.02	21.52 (2,1818.3)	*p* < 0.001^b^ ^,^ ^c^	0.560/0.016	0.02
	Minimalist	43.14 ± 9.11	43.89 ± 8.81								
	Barefoot	39.98 ± 8.74	42.03 ± 8.94								
Anticipation				14.47 (1,1813.3)	*p* < 0.001		0.008	5.05 (1,1829.0)	*p* = 0.025		0.003
Approach velocity								3.07 (1,1851.2)	*p* = 0.080		0.002

^a^ Post hoc test significantly different (*p* < 0.05) between standard sport shoes and minimalist shoes.

^b^ Post hoc test significantly different (*p* < 0.05) between standard sport shoes and barefoot.

^c^ Post hoc test significantly different (*p* < 0.05) between minimalist shoes and barefoot.

^d^ ANOVA significant interaction effect (*p* < 0.05) between anticipation and footwear.

**FIGURE 8:**
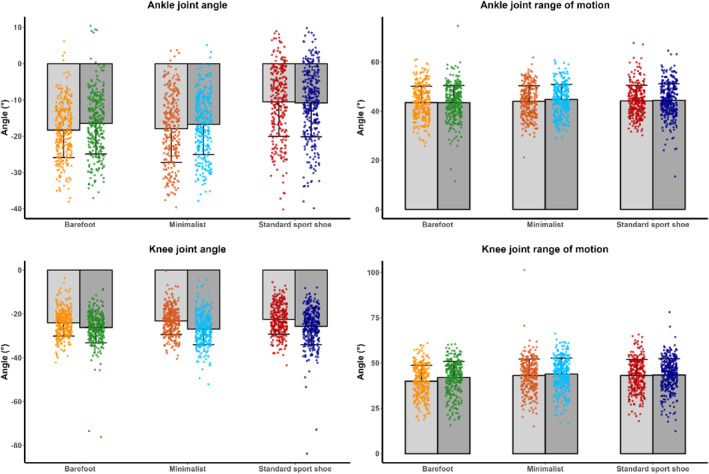
Mean and standard deviation of initial joint angle (left) and the range of motion (right) for the ankle and knee joints across footwear conditions, presented as scatterplots combined with bar plots. Warm colors represent the anticipated condition, and cool colors represent the unanticipated condition. Point clouds show individual trial data in their respective colors. Mean and standard deviation are illustrated by bars, with gray bars indicating the anticipated and dark gray bars the unanticipated condition. Color coding is as follows: standard sport shoe—red (anticipated) and dark blue (unanticipated), minimalist shoe—orange (anticipated) and cyan (unanticipated), and barefoot—yellow (anticipated) and green (unanticipated).

Without covariate adjustment, footwear had a small but significant effect on the knee joint angle at initial contact (*p* < 0.001), with standard and minimalist shoes showing less knee flexion than barefoot. However, this effect disappeared after controlling for approach velocity (*p* = 0.136). Conversely, anticipation consistently influenced the knee joint angle (*p* < 0.001). For knee ROM, both footwear and anticipation significantly increased ROM (*p* < 0.001) in both unadjusted and adjusted models, with standard and minimalist shoes showing greater knee ROM than barefoot (Figure 8).

#### 3.1.3 Muscle Activity

Footwear significantly influenced all muscle activity parameters (*p* < 0.01), except peak TA activity. Standard sport shoes led to higher or comparable peak GL and GM activation than minimalist or barefoot condition. Although anticipation had no clear effect, approach velocity significantly influenced GM and TA in the adjusted model (*p* < 0.05). TA showed no footwear effect on peak activity until approach velocity was included as a covariate (*p* < 0.05).

GL and GM showed a significant footwear effect on mean muscle activity (*p* < 0.001), with standard and minimalist shoes showing high mean activity. Anticipation significantly increased mean activity (*p* < 0.01) for GL and TA. TA mean activation was significantly influenced by footwear (*p* < 0.001); anticipation had a small effect. Approach velocity demonstrated significant influence on GL and TA activity, accompanied by negligible variations in the *p* values of the adjusted model in all muscles (Table 5).

**TABLE 5:** ANOVA results for sEMG parameters—effects of footwear and anticipation with and without approach velocity as a covariate.

		Anticipation		ANOVA without covariate				ANOVA with covariate			
		With	Without	F (df1, df2)	*p* value	Conditional/marginal *R* ^2^	*η* ^2^	F (df1, df2)	*p* value	Conditional/marginal *R* ^2^	*η* ^2^
Peak muscle activity of the gastrocnemius lateralis [uV]											
Footwear	Standard	4.40e‐04 ± 2.57e‐04	4.58e‐04 ± 2.68e‐04	23.05 (2,1813.7)	*p* < 0.001^b^ ^,^ ^c^	0.622/0.010	0.02	20.34 (2,1816.7)	*p* < 0.001^b^ ^,^ ^c^	0.623/0.010	0.02
	Minimalist	4.36e‐04 ± 2.64e‐04	4.33e‐04 ± 2.69e‐04								
	Barefoot	4.02e‐04 ± 2.39e‐04	3.93e‐04 ± 2.43e‐04								
Anticipation				1.74 (1,1813.1)	*p* = 0.187		0.001	0.83 (1,1825.4)	*p* = 0.363		0.0005
Approach velocity								0.13 (1,1847.3)	*p* = 0.716		7E‐05
Peak muscle activity of the gastrocnemius medialis [uV]^d^											
Footwear	Standard	3.88e‐04 ± 1.91e‐04	4.06e‐04 ± 2.23e‐04	6.89 (2,1813.5)	*p* = 0.001^b^ ^,^ ^c^	0.641/0.004	0.008	3.54 (2,1816.3)	*p* = 0.029^c^	0.637/0.007	0.004
	Minimalist	3.96e‐04 ± 2.04e‐04	4.06e‐04 ± 2.40e‐04								
	Barefoot	3.89e‐04 ± 2.00e‐04	3.61e‐04 ± 1.90e‐04								
Anticipation				0.03 (1,1812.9)	*p* = 0.859		0.00002	2.42 (1,1824.5)	*p* = 0.120		0.001
Approach velocity								6.71 (1,1846.1)	*p* = 0.010		0.004
Peak muscle activity of the tibialis anterior [uV]											
Footwear	Standard	3.33e‐04 ± 1.61e‐04	3.02e‐04 ± 1.33e‐04	1.18 (2,1814.3)	*p* = 0.309	0.501/0.013	0.001	5.83 (2,1819.8)	*p* = 0.003^b^ ^,^ ^c^	0.507/0.029	0.006
	Minimalist	3.32e‐04 ± 1.72e‐04	2.94e‐04 ± 1.45e‐04								
	Barefoot	3.37e‐04 ± 1.47e‐04	3.08e‐04 ± 1.37e‐04								
Anticipation				45.55 (1,1813.3)	*p* < 0.001		0.02	7.67 (1,1832.7)	*p* = 0.006		0.004
Approach velocity								29.67 (1,1851.7)	*p* < 0.001		0.02
Mean muscle activity of the gastrocnemius lateralis [uV]											
Footwear	Standard	1.65e‐04 ± 8.94e‐05	1.83e‐04 ± 9.72e‐05	60.21 (2,1813.7)	*p* < 0.001^b^ ^,^ ^c^	0.664/0.027	0.06	48.54 (2,1816.2)	*p* < 0.001^b^ ^,^ ^c^	0.662/0.028	0.05
	Minimalist	1.69e‐04 ± 9.11e‐05	1.79e‐04 ± 1.03e‐04								
	Barefoot	1.49e‐04 ± 7.53e‐05	1.47e‐04 ± 7.58e‐05								
Anticipation				20.53 (1,1813.2)	*p* < 0.001		0.01	18.58 (1,1823.9)	*p* < 0.001		0.01
Approach velocity								0.87 (1,1844.7)	*p* = 0.351		0.0005
Mean muscle activity of the gastrocnemius medialis [uV]^d^											
Footwear	Standard	1.58e‐04 ± 8.66e‐05	1.70e‐04 ± 9.45e‐05	23.82 (2,1813.5)	*p* < 0.001^a^ ^,^ ^b^ ^,^ ^c^	0.725/0.008	0.03	20.04 (2,1815.2)	*p* < 0.001^a^ ^,^ ^c^	0.723/0.014	0.02
	Minimalist	1.76e‐04 ± 9.54e‐05	1.76e‐04 ± 8.97e‐05								
	Barefoot	1.62e‐04 ± 7.91e‐05	1.52e‐04 ± 7.74e‐05								
Anticipation				0.19 (1,1813.1)	*p* = 0.663		0.0001	7.00 (1,1821.0)	*p* = 0.008		0.004
Approach velocity								17.57 (1,1838.6)	*p* < 0.001		0.009
Mean muscle activity of the tibialis anterior [uV]											
Footwear	Standard	1.57e‐04 ± 6.91e‐05	1.42e‐04 ± 6.45e‐05	7.56 (2,1813.8)	*p* < 0.001^c^	0.626/0.016	0.008	19.82 (2,1816.9)	*p* < 0.001^b^ ^,^ ^c^	0.639/0.040	0.02
	Minimalist	1.52e‐04 ± 7.49e‐05	1.37e‐04 ± 6.79e‐05								
	Barefoot	1.63e‐04 ± 6.90e‐05	1.47e‐04 ± 6.83e‐05								
Anticipation				63.61 (1,1813.2)	*p* < 0.001		0.03	6.52 (1,1825.3)	*p* = 0.011		0.004
Approach velocity								61.32 (1,1847.0)	*p* < 0.001		0.03
Muscle activity of gastrocnemius lateralis at initial ground contact [uV]^d^											
Footwear	Standard	1.17e‐04 ± 1.26e‐04	1.38e‐04 ± 1.38e‐04	39.75 (2,1815.2)	*p* < 0.001^a^ ^,^ ^b^	0.418/0.027	0.04	42.35 (2,1822.8)	*p* < 0.001^a^ ^,^ ^b^	0.419/0.030	0.04
	Minimalist	1.77e‐04 ± 1.53e‐04	1.75e‐04 ± 1.47e‐04								
	Barefoot	1.88e‐04 ± 1.58e‐04	1.72e‐04 ± 1.30e‐04								
Anticipation				0.50 (1,1813.8)	*p* = 0.481		0.0003	3.54 (1,1839.1)	*p* = 0.060		0.002
Approach velocity								5.68 (1,1840.1)	*p* = 0.017		0.003
Muscle activity of gastrocnemius medialis at initial ground contact [uV]^d^											
Footwear	Standard	1.37e‐04 ± 1.10e‐04	1.56e‐04 ± 1.51e‐04	70.50 (2,1815.3)	*p* < 0.001^a^ ^,^ ^b^ ^,^ ^c^	0.399/0.048	0.07	86.28 (2,1824.1)	*p* < 0.001^a^ ^,^ ^b^ ^,^ ^c^	0.405/0.067	0.09
	Minimalist	1.99e‐04 ± 1.36e‐04	1.97e‐04 ± 1.33e‐04								
	Barefoot	2.27e‐04 ± 1.46e‐04	2.02e‐04 ± 1.30e‐04								
Anticipation				0.38 (1,1813.7)	*p* = 0.538		0.0002	5.83 (1,1842.0)	*p* = 0.016		0.003
Approach velocity								29.75 (1,1826.9)	*p* < 0.001		0.02
Muscle activity of tibialis anterior at initial ground contact [uV]^d^											
Footwear	Standard	1.70e‐04 ± 1.13e‐04	1.57e‐04 ± 1.11e‐04	40.34 (2,1815.6)	*p* < 0.001^a^ ^,^ ^b^	0.318/0.034	0.04	24.54 (2,1828.4)	*p* < 0.001^a^ ^,^ ^b^ ^,^ ^c^	0.316/0.073	0.03
	Minimalist	1.31e‐04 ± 9.68e‐05	1.16e‐04 ± 8.84e‐05								
	Barefoot	1.34e‐04 ± 8.92e‐05	1.23e‐04 ± 8.75e‐05								
Anticipation				12.31 (1,1813.4)	*p* < 0.001		0.007	0.78 (1,1849.5)	*p* = 0.378		0.0004
Approach velocity								52.76 (1,1736.5)	*p* < 0.001		0.03

^a^ Post hoc test significantly different (*p* < 0.05) between standard sport shoes and minimalist shoes.

^b^ Post hoc test significantly different (*p* < 0.05) between standard sport shoes and barefoot.

^c^ Post hoc test significantly different (*p* < 0.05) between minimalist shoes and barefoot.

^d^ ANOVA significant interaction effect (*p* < 0.05) between anticipation and footwear.

**FIGURE 9:**
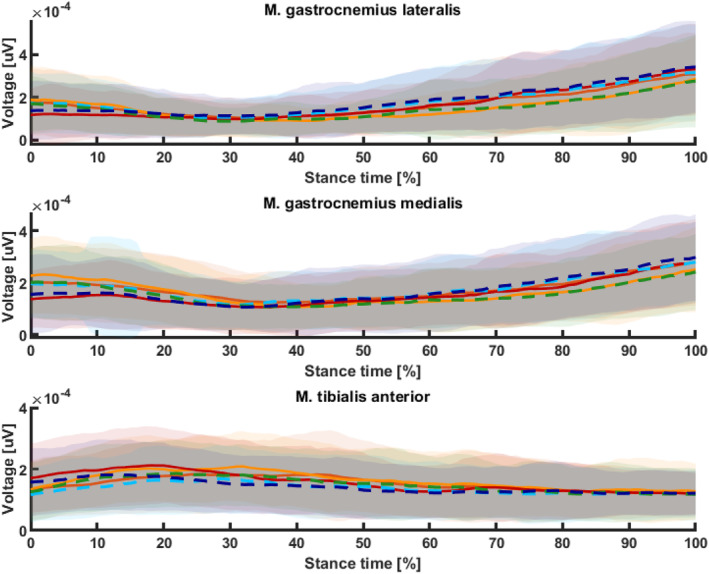
Interpolated mean and standard deviation of EMG curves during the stance phase of the 90° change‐of‐direction task across footwear and anticipation conditions. Warm‐colored solid lines represent the anticipated condition, and cool‐colored dashed lines represent the unanticipated condition. The standard sport shoe condition is shown in red and dark blue, the minimalist shoe condition in orange‐red and cyan, and the barefoot condition in orange and green.

At initial ground contact, barefoot and minimalist shoes showed both significantly higher muscle activity for GM and GL than standard sport shoe (*p* < 0.001), whereas the TA activity was the highest in standard sport shoe (*p* < 0.001). Anticipation significantly influenced only initial ground contact TA activity (*p* < 0.001), with reduced sEMG amplitude in the unanticipated condition. In the adjusted model, GM anticipation effects became significant, whereas TA effects became nonsignificant. Approach velocity significantly affected all muscles (Figure 9).

## 4 Discussion

In accordance with our hypothesis, the results of this study demonstrate that performance during anticipated and unanticipated 90° COD is significantly influenced by the type of footwear worn. Given the significant differences in approach velocity, a linear mixed model including approach velocity as a covariate was additionally conducted. However, the footwear effect remained significant for nearly all variables. The observed alterations in kinematics, kinetics, and muscle activity highlight how athletes adapt their movement strategies in response to different footwear conditions.

### 4.1 Spatiotemporal Characteristics

In particular, the reduction in approach and toe‐off velocity, as well as the altered exit angle, indicate a modified movement strategy with minimalist footwear and barefoot condition, which negatively impacted performance. As approach velocity is a major factor influencing COD performance [38] and joint loading [47], previous studies have standardized this variable (e.g., 4 m/s ± 5%–10%) to ensure comparability across conditions [23, 24, 48, 49, 50]. In contrast, the present study allowed natural variations in approach behavior and performance changes driven by the manipulation of footwear conditions.

The results suggest that movement adjustments likely occurred during the approach phase leading up to the final step of the COD, as participants in the minimalist and barefoot condition appeared to decelerate earlier or more strongly, resulting in lower pre‐COD velocity and a shallower exit angle than standard sport shoes. Since COD is a multistep movement [51], braking forces are distributed across multiple foot contacts, with the penultimate foot contact playing a key role in deceleration by absorbing a significant portion of the braking forces and controlling momentum before the final foot contact [52, 53, 54]. The observed differences in approach velocity and movement execution suggest that minimalist and barefoot condition led to meaningful adjustments in the (sub)conscious planning or programming of the challenging task. Although the reasons remain unknown, unfamiliarity may have heightened awareness of potential risks (e.g., changes in cushioning or traction), triggering a more robust braking strategy.

Additionally, barefoot condition resulted in shorter ground contact times (GCT), braking, and propulsion phases than standard sport shoes. Minimalist footwear had the longest propulsion but a shorter braking phase than standard shoes. Participants in barefoot and minimalist conditions accelerated during the final stance phase, whereas velocity remained stable in standard shoes. From a biomechanical perspective, the impulse required to change direction depends on approach velocity and momentum transfer, with impulse being the product of force and time, directly influencing changes in momentum. Since approach velocity was lower in the barefoot and minimalist conditions, the required braking impulse was smaller, resulting in shorter braking times and GCT. However, the differences in GCT, braking, and propulsion time remained significant in the covariate model, indicating a specific footwear effect beyond approach velocity. One possible mechanism, also observed in running research [55, 56], could be neuromuscular adjustments, as reflected by increased leg stiffness in the barefoot condition, quantified through significantly lower knee and ankle joint ROM than both shod conditions.

### 4.2 Lower Extremity Kinematics

In running research, harder surfaces or uncushioned footwear often shift foot strike toward FFS with greater knee flexion [57, 58], facilitating impact absorption through the posterior calf muscles [21, 59]. In our study, moving to uncushioned footwear induced a similar shift toward FFS and MFS, as indicated by the center of pressure at initial contact and significant changes in ankle joint kinematics, characterized by greater plantarflexion at ground contact and increased ankle range of motion compared to the standard shoe condition, with large effect sizes. We also observed a slightly more flexed knee joint at ground contact in the barefoot and minimalist conditions, although this effect became nonsignificant once approach velocity was accounted for. Although roughly half of all anticipated 90° CODs in standard sport shoes were executed with an RFS and about a quarter with FFS, the proportion of FFS doubled in minimalist and barefoot condition, reducing RFS to 25% and 15%, respectively. These differences were mirrored in joint angles: standard shoes led to greater ankle dorsiflexion and knee extension at initial contact than minimalist or barefoot condition. Such kinematic adjustments are consistent with earlier observations that footwear cushioning influences foot strike strategy and joint positioning. Despite these parallels, footwear‐related changes in COD kinematics remain underexplored. Sinclair et al. [23] investigated 45° COD but noted no significant differences in peak joint angles, implying that minimalist footwear did not substantially alter movement kinematics under those conditions. Conversely, Bisesti et al. [48] reported that athletes performing a 45° cutting maneuver barefoot used a more anterior foot strike than those wearing standard shoes, underscoring the influence of footwear choice on FSP. Similarly, Sinclair et al. [24] examined 180° COD and found a more plantarflexed ankle in minimalist shoes. These discrepancies may be explained by differences in the tested cutting angles [26] and the specific footwear models [15, 16, 17] used, which likely affected the movement demands and resulting kinematic adaptations.

### 4.3 Muscle Activity

Calf muscle activity adapts to different footwear conditions. Although the overall shape of the sEMG curve remains relatively constant throughout the stance phase, the amplitude of muscle activation varies depending on the type of footwear and the phase of stance. In the early stance phase, plantarflexor activity is higher among minimalist and barefoot runners, whereas TA activity is greater in standard sport shoes. Differences between footwear conditions also tend to emerge toward the end of the stance phase in the plantarflexors, with both shoe conditions showing higher activity than the barefoot condition, indicating higher propulsion activity. In contrast, in the TA, these differences diminish and are no longer prominent after the mid‐stance phase.

Likewise, the sEMG signal of the plantarflexors at initial contact is increased under barefoot and minimalist conditions, indicating pre‐activation and adaptation of the FSP. These results are in line with the findings of Yoshida et al. [60], who investigated muscle activity in 45° COD manipulating FSP. The authors concluded that FFS during COD was associated with lower knee injury and risk muscle activity, as plantarflexor and biceps’ femoris activity were significantly higher during FFS COD. However, this difference in load distribution results in increased stress on other structures, such as the achilles tendon.

Peak and mean muscle activity mainly differs in the plantarflexors, with significant differences observed only between the two shoe conditions and the barefoot condition. The activity of TA and GM was significantly influenced by approach velocity. Despite altered kinematics, both peak and mean activity of the GL were highest in the standard sport shoe, whereas GM showed slightly lower activity. This effect may, at least in part, be attributed to approach velocity [61]. Interestingly, peak and mean TA activity were highest in barefoot condition. This is noteworthy, as FFS would typically be expected to result in lower TA activity. However, this increased activation may indicate a co‐contraction response to stabilize the unfamiliar barefoot condition [59].

### 4.4 Kinetics

Participants generated the highest forces in all directions with standard sport shoes, particularly in the anterior–posterior and medial‐lateral directions, which are critical for effective braking, reacceleration, and stabilization during COD movements. Compared to standard shoes, minimalist and barefoot condition reduced maximum braking force in the anterior–posterior direction by ∼15% and 30%, and maximum propulsion force in the medial–lateral direction by ∼5% and 10%, respectively. In almost all variables, the footwear effect remained significant even when approach velocity was included as a covariate, with moderate to high effect sizes, especially in the medial–lateral and anterior–posterior directions. In contrast, vertical forces exhibited small effect sizes, suggesting only minor relevant changes that are movement‐dependent, as vertical forces’ play are more relevant in running than COD [62].

Higher force generation in anterior–posterior and medial–lateral directions enhances momentum transfer, improving deceleration and reacceleration [63]. This advantage, which contributed to the superior performance observed in the standard sport shoe condition, may be attributed to specific footwear properties. Features such as shoe mass [64], forefoot bending stiffness [65, 66], outsole traction [66, 67], and upper configuration [68], have been shown to influence biomechanics and enhance performance. Notably, Worobets et al. [66] found that a 20% reduction in traction impaired COD performance by up to 30%, although further increases in the dynamic friction coefficient beyond an optimal threshold did not provide additional performance benefits [67]. Greater traction allows athletes to lean more into the surface, directing the GRF more effectively in the desired direction, potentially increasing horizontal GRF [67, 69]. In our analysis, the DFC reflects the required or utilized traction (friction utilization) derived from GRF, rather than the available traction of the footwear–surface combination. DFC values in our study (∼0.5–0.6) fall within published ranges for COD tasks (0.41–1.07) [70]. Because DFC depends on horizontal force production, the lower approach velocity and reduced anterior–posterior/medial–lateral forces in the minimalist and barefoot condition likely contributed to lower utilized traction in those conditions. Accordingly, while we observed ∼10% and 3.6% higher DFC in standard shoes relative to barefoot and minimalist conditions, these differences should be interpreted primarily as evidence of a more aggressive braking/reacceleration strategy rather than as direct indicators of superior outsole traction. This interpretation is supported by Fong et al. [5], who noted that reduced traction availability or perceived grip can lead to more cautious gait patterns, consistent with the conservative approach observed in the minimalist and barefoot condition. Nevertheless, since only utilized traction was quantified and the specific mechanical traction properties of the footwear–surface combinations were not directly assessed, these interpretations remain speculative.

Vertical load, a potential running injury risk indicator [71, 72, 73], showed a significant footwear effect with a small effect size, suggesting sensitivity to COD conditions. Vertical instantaneous loading was lower in standard sport shoes, likely due to their cushioning properties, which dampen the initial passive phase of ground contact. In contrast, vertical average loading was lowest in barefoot and highest in standard shoes, indicating distinct loading patterns. Notably, a change in FSP is often associated with the absence or reduction of an impact peak in the force–time curve, which may explain the lower average loading observed in barefoot and minimalist conditions. Sinclair et al. also reported higher vertical loading rates in 180° and 45° cutting maneuvers. However, unlike Sinclair [24], we found significantly lower average loading rates in barefoot and minimalist shoes than in standard shoes, even after controlling for approach velocity. Although Sinclair [24] used a standardized approach velocity, exact values were not reported; only a 5% deviation criterion was reported. Our velocity deviations were similarly within ∼5% of the minimalist shoe mean, ruling out approach velocity as a confounding factor. Thus, the discrepancies in results are likely due to differences in shoe models and the COD angle.

### 4.5 Anticipation

Recent meta‐analysis by Ebner et al. [74] demonstrated that unanticipated COD lead to greater knee flexion angles during stance than anticipated movements, highlighting the importance of including anticipation as an experimental factor. This study is the first to explore how different footwear conditions interact with unanticipated COD, mirroring the rapid reactions required in game‐like scenarios. Limited preparation time inherent to unanticipated tasks substantially alters biomechanics and performance, with increased neuromuscular demands (elevated sEMG values), despite the reduction in velocity. However, we did not observe meaningful interactions between anticipation and footwear: although unanticipated movements differed from anticipated ones in terms of spatiotemporal parameters and biomechanical measures, similar to previous studies [49, 50], these differences did not vary substantially by footwear. Therefore, although unanticipated movements generally showed reduced performance time, lower approach velocity, reduced kinetic demands, and small kinematic differences, the overall effect of footwear remained consistent regardless of anticipation level.

### 4.6 Practical Implications

Studies that manipulated FSP during COD tasks have demonstrated positive effects on high‐risk knee joint kinematics and kinetics at initial ground contact [10, 11]. These include reductions in knee valgus, tibial internal rotation moments, and less extended knee joint angles/moments—factors associated with injuries such as ACL ruptures [75, 76, 77]. Selecting appropriate footwear could provide a natural means of influencing FSP. However, to optimize performance in minimalist shoes, it is crucial to consider both short‐term technique adaptations and long‐term benefits. For instance, peak ankle power, peak ankle plantarflexor moment, and shorter GCT have been strongly correlated with improved COD performance. Minimalist shoes, though challenging initially, could strengthen foot musculature over time, particularly the toe flexors, as demonstrated by Goldman et al. [78]. This strengthening may lead to enhanced performance in COD tasks in the long term. To further enhance performance in minimalist shoes, manufacturers should consider optimizing shoe properties to better meet biomechanical demands. For example, incorporating improved traction elements into the forefoot area of the outsole could better support forefoot strikes during COD tasks and facilitate effective force application.

### 4.7 Limitations

This study has several limitations. Slight differences in heel‐to‐toe offset may have influenced the neutral ankle alignment between shoes. This effect is unlikely to affect the interpretation of between‐condition differences but should be considered when comparing absolute ankle angles. Although the participants' habitual footwear was not standardized, which may have introduced variability and served as a potential confounding factor, this approach enhances ecological validity by reflecting real‐world conditions in which athletes use their own footwear. In addition, only the final foot contact during the COD was analyzed, although the penultimate contact also plays a critical role in deceleration and movement preparation [52], which may limit the completeness of the biomechanical interpretation. All participants were habitual shod individuals engaged in nonbarefoot sports, limiting generalizability to populations accustomed to minimalist footwear, barefoot running, or barefoot sports like martial arts. Additionally, unfamiliarity with minimalist or barefoot condition may have influenced results, emphasizing the need for long‐term studies to determine if initial adaptations persist. The tests were conducted in a controlled laboratory setting, ensuring standardization but limiting applicability to real‐world surfaces like grass or turf. As this study focused on acute footwear effects, long‐term habituation could alter performance and biomechanical responses. Additionally, participants' habitual sport shoes were used as the standard condition, introducing variability in shoe properties like cushioning, whereas minimalist and barefoot condition were standardized. Thus, results for standard shoes should be interpreted cautiously. Lastly, this study did not directly measure the material properties of the footwear or the surface, limiting attributions of observed differences to specific characteristics. Future research should incorporate direct measurements of footwear and surface properties and expand testing to include various surface types to better understand surface–footwear interactions.

## 5 Conclusion

This study underscores the significant influence of footwear on biomechanics, performance, and neuromuscular strategies during 90° COD tasks. Standard sport shoes supported higher velocities and better overall performance, whereas minimalist and barefoot condition introduced distinct foot strike patterns and joint kinematics that have been associated in previous studies with reduced ACL loading and altered knee joint moments. Although our study did not directly assess injury risk, these adaptations could theoretically contribute to a more favorable load distribution across the lower limb. Despite changes in movement strategy for unanticipated CODs, overall footwear effects remained stable. These findings emphasize the role of footwear in balancing performance and factors related to injury prevention, providing a foundation for future research on long‐term adaptations, footwear design, and surface interactions.

## Author Contributions

**Stanislav Dimitri Siegel:** investigation (lead), formal analysis (lead), methodology (lead), project administration (lead), software (lead), visualization (lead), data curation (lead), writing – original draft (lead), writing – review and editing (equal). **Mareike Sproll:** writing – review and editing (supporting), validation (supporting), investigation (supporting), visualization (supporting). **Konstantin Warneke:** writing – review and editing (equal), validation (supporting). **Joel Mason:** writing – review and editing (equal). **Astrid Zech:** conceptualization (lead), project administration (supporting), resources (lead), supervision (lead), writing – review and editing (equal).

## Funding

Leguano GmbH has provided financial and material resources for the data collection.

## Ethics Statement

Ethical approval was obtained from the Ethics Committee of the Faculty of Social and Behavioural Sciences of the Friedrich Schiller University Jena (protocol number FSV 21/003). During the study process, the authors followed the rules of the Helsinki Declaration.

## Consent

All participants were informed of the potential risks, benefits, and dissemination of the research before providing written informed consent to participate. Furthermore, all participants and, in the case of minors, their parents/legal guardians, gave their written consent to participate in this study and were informed that they could withdraw their participation at any time without giving reasons.

## Conflicts of Interest

The authors declare no conflicts of interest.

## Supporting information


Supporting Information S1

## Data Availability

The raw datasets generated during and/or analyzed during the current study are not publicly available.
