# Supplementary material for: Acute Change of Footwear Limits Performance and Alters Foot Strike Patterns During Anticipated and Unanticipated 90° Change of Direction
Source: J Foot Ankle Res. 2025 Dec 3;18(4):e70103. doi: 10.1002/jfa2.70103 (PMC12675306; doi:10.1002/jfa2.70103)
Supplement: Supplementary file 1 — Supporting Information S1 [file JFA2-18-e70103-s001.docx]

# Appendix

Table 1: Shoe type, brand and model of each subject standard sport shoes

| **Participant** | **Shoe type** | **Brand** | **Modell** |
| --- | --- | --- | --- |
| 1 | Indoor shoe | Adidas | Sala |
| 2 | Casual shoe | Lico | Limber |
| 3 | Indoor shoe | Nike | Jordan Westbrook One Take |
| 4 | Running shoe | Evadict | Trail XT7 |
| 5 | Running shoe | Asics | GEL-Fuji Attack 2 |
| 6 | Running shoe | Adidas | Ultraboost 20 |
| 7 | Indoor shoe | Yonex | Badmintonschuh |
| 8 | Running shoe | New Balance | Fresh Foam |
| 9 | Running shoe | Asics | Gel Cumulus 17 |
| 10 | Indoor shoe | Adidas | Hb Special Pro |
| 11 | Running shoe | Adidas | NMD |
| 12 | Running shoe | Hoka | One One Torrent |
| 13 | Running shoe | Adidas | Alphabounce |
| 14 | Running shoe | Puma | NRGY Star Multiknit |
| 15 | Running shoe | Brooks | Ravenna |
| 16 | Running shoe | Nike | Flex Trainer |
| 17 | Running shoe | Brooks | Ghost 13 |
| 18 | Running shoe | Nike | Legend react 2 AT |
| 19 | Running shoe | Adidas | Ultraboost |
| 20 | Running shoe | Adidas | Ultraboost |
| 21 | Running shoe | Nike | Renew ride |
| 22 | Running shoe | Nike | Air Zoom Vomero |
| 23 | Running shoe | Adidas | Ultraboost |
| 24 | Running shoe | Nike | Air Zoom Terra Kiger |
| 25 | Running shoe | Nike | Legend react |
| 26 | Running shoe | Adidas | Adizero Adios 5 |
| 27 | Running shoe | Brooks | Glycerin |
| 28 | Indoor shoe | Nike | Tiempo Mystic IV |
| 29 | Running shoe | Adidas | Ultraboost |
| 30 | Running shoe | Nike | Legend react |
| 31 | Casual shoe | Nike | Air Force |
| 32 | Running shoe | Adidas | Ultraboost |
| 33 | Running shoe | Nike | Flex Trainer |
| 34 | Running shoe | Yonex | Power Cushion |
| 35 | Running shoe | Nike | Lunargato |
| 36 | Running shoe | Asics | Gel-Pulse |
| 37 | Running shoe | Nike | Legend react |
| 38 | Running shoe | Nike | epic react flyknit |
| 39 | Running shoe | Adidas | Ultraboost |
| 40 | Running shoe | Nike | Performance Revolution |
| 41 | Running shoe | Nike | Mercurial Vapor |

Table 2 Definition of variables used in the 90° cutting maneuver analysis

| **Variable** | **Definition** |  |
| --- | --- | --- |
| **Performance time [s]** | Time to complete cutting task |  |
| **Approach velocity [m*s^-1^]** | Mean horizontal velocity calculated from sacrum position data over the five frames before ground contact [10] |  |
| **Toe-off velocity [m*s^-1^]** | Horizontal velocity at toe-off calculated from sacrum position data. |  |
| **Change of direction angle [°]** | The absolute value of the angle (in degrees) between the x-velocity and y-velocity of the virtual sacrum marker at the toe-off frame position [38] |  |
| **Ground contact time [s]** | Time difference between toe-off and ground contact |  |
| **Braking time [s]** | Time from initial ground contact until maximum knee flexion angle |  |
| **Propulsion time [s]** | Time from maximum knee flexion angle to toe-off |  |
| **Vertical instantaneous load [BW*s^-1^]** | Maximum instantaneous slope of the vertical load from 3-12% of stance phase [79] |  |
| **Vertical average load [BW*s^-1^]** | Average slope of the vertical load from 3-12% of stance phase [79] |  |
| **Maximum force during the braking phase [BW]** | Maximum force in the vertical, posterior-anterior or medio-lateral direction during the braking phase |  |
| **Maximum force during the propulsion phase [BW]** | Maximum force in the vertical, posterior-anterior or medio-lateral direction during the propulsion phase |  |
| **Mean force [BW]** | | Mean force in the vertical, posterior-anterior or medio-lateral direction during the stance phase |
| **Dynamic friction coefficient** | Square root of the sum of the squares of the forces in the X and Y directions, and then dividing by the force in the Z direction[70]. We calculated the mean across the stance phase. |  |
| **Center of pressure distance [m]** | Euclidean distances between consecutive COP points during the stance phase |  |
| **Ankle joint angle [°]** | Sagittal plane ankle angle at initial ground contact |  |
| **Ankle joint range of motion [°]** | Range of motion of the ankle joint in the sagittal plane during the stance phase  (maximum angle minus the minimum angle) |  |
| **Knee joint angle [°]** | | Sagittal plane knee angle at initial ground contact |
| **Knee joint range of motion [°]** | | Range of motion of the knee joint in the sagittal plane during the stance phase (maximum angle minus the minimum angle) |
| **Peak muscle activity [mV]** | Peak EMG value of the corresponding muscle during the stance phase |  |
| **Mean muscle activity [mV]** | Mean EMG value of the corresponding muscle during the stance phase |  |

*Abbreviations:* **BW** = body weight; **EMG** = electromyography, **iEMG** = integrated electromyograph

Table 3: Fixed Effects Estimates from Linear Mixed Models with and without Covariate for all variables

|  | | **Without Covariate** | | | | | **With Covariate** | | | | |
| --- | --- | --- | --- | --- | --- | --- | --- | --- | --- | --- | --- |
| **Fixed Effects** | | ***β*** | **SE** | **t** | **p** | **95% CI** | ***β*** | **SE** | **t** | **p** | **95% CI** |
| **Performance time [s]** | |  |  |  |  |  |  |  |  |  |  |
| (Intercept) | | 1.63 | 0.015 | 105.89 | <0.001 | 1.60-1.66 | 2.07 | 0.03 | 70.9 | <0.001 | 2.01-2.13 |
| Barefoot vs. minimalist | | 0.04 | 0.01 | 4.86 | <0.001 | 0.02-0.06 | 0.02 | 0.01 | 2.36 | 0.018 | 0.003-0.03 |
| Standard vs. minimalist | | -0.04 | 0.01 | -4.47 | <0.001 | -0.05 - -0.02 | -0.02 | 0.01 | -2.39 | 0.017 | -0.03- -0.003 |
| Anticipation (No vs. Yes) | | 0.14 | 0.01 | 16.84 | <0.001 | 0.12-0.16 | 0.1 | 0.01 | 11.5 | <0.001 | 0.08-0.11 |
| Approach Velocity (Covariate) | |  |  |  |  |  | -0.14 | 0.01 | -16.53 | <0.001 | -0.16 - -0.12 |
| Interaction barefoot/minimalist x Anticipation | | 0.02 | 0.01 | 1.35 | 0.177 | ‚-0.007-0.04 | 0.01 | 0.01 | 1.21 | 0.225 | ‚-0.01-0.04 |
| Interaction standard/minimalist x Anticipation | | 0.001 | 0.01 | 0.09 | 0.929 | ‚-0.02-0.02 | -0.004 | 0.01 | -0.34 | 0.738 | ‚-0.03-0.02 |
| **Approach velocity [m*s-1]** | |  |  |  |  |  |  |  |  |  |  |
| (Intercept) | | 3.16 | 0.05 | 69.55 | <0.001 | 3.07-3.25 | 3.16 | 0.05 | 69.55 | <0.001 | 3.07-3.25 |
| Barefoot vs. minimalist | | -0.16 | 0.02 | -7.3 | <0.001 | -0.20- -0.12 | -0.16 | 0.02 | -7.3 | <0.001 | -0.20- -0.12 |
| Standard vs. minimalist | | 0.13 | 0.02 | 6.21 | <0.001 | 0.09-0.18 | 0.13 | 0.02 | 6.21 | <0.001 | 0.09-0.18 |
| Anticipation (No vs. Yes) | | -0.33 | 0.02 | -15.19 | <0.001 | -0.37- -0.28 | -0.33 | 0.02 | -15.19 | <0.001 | -0.37- -0.28 |
| Approach Velocity (Covariate) | |  |  |  |  |  |  |  |  |  |  |
| Interaction barefoot/minimalist x Anticipation | | -0.02 | 0.03 | -0.58 | 0.561 | ‚-0.08-0.04 | -0.02 | 0.03 | -0.58 | 0.561 | ‚-0.08-0.04 |
| Interaction standard/minimalist x Anticipation | | -0.03 | 0.03 | -1.12 | 0.262 | ‚-0.09-0.03 | -0.03 | 0.03 | -1.12 | 0.262 | ‚-0.09-0.03 |
| **Toe-off velocity [m*s-1]** | |  |  |  |  |  |  |  |  |  |  |
| (Intercept) | | 3.20 | 0.03 | 98.33 | <0.001 | 3.13-3.26 | 2.29 | 0.07 | 32.94 | <0.001 | 2.15-2.42 |
| Barefoot vs. minimalist | | -0.06 | 0.02 | -2.83 | 0.005 | -0.10- -0.02 | -0.01 | 0.02 | -0.54 | 0.587 | -0.05-0.03 |
| Standard vs. minimalist | | 0.09 | 0.02 | 4.68 | <0.001 | 0.05-0.13 | 0.05 | 0.02 | 2.83 | 0.005 | 0.02-0.09 |
| Anticipation (No vs. Yes) | | -0.15 | 0.02 | -7.54 | <0.001 | -0.19- -0.11 | -0.05 | 0.02 | -2.72 | 0.007 | -0.09- -0.02 |
| Approach Velocity (Covariate) | |  |  |  |  |  | 0.29 | 0.02 | 14.49 | <0.001 | 0.25-0.33 |
| Interaction barefoot/minimalist x Anticipation | | 0.01 | 0.03 | 0.37 | 0.710 | ‚-0.04-0.06 | 0.02 | 0.03 | 0.59 | 0.556 | ‚-0.04-0.07 |
| Interaction standard/minimalist x Anticipation | | -0.02 | 0.03 | -0.56 | 0.576 | ‚-0.07-0.04 | -0.01 | 0.03 | -0.22 | 0.829 | ‚-0.06-0.05 |
| **Change of direction angle [°]** | |  |  |  |  |  |  |  |  |  |  |
| (Intercept) | | 52.62 | 0.68 | 77.54 | <0.001 | 51.28-53.96 | 69.05 | 1.61 | 42.96 | <0.001 | 65.91-72.20 |
| Barefoot vs. minimalist | | -1.79 | 0.46 | -3.91 | <0.001 | -2.68- -0.89 | -2.61 | 0.45 | -5.83 | <0.001 | -3.49- -1.74 |
| Standard vs. minimalist | | 1.87 | 0.46 | 4.11 | <0.001 | 0.98-2.77 | 2.58 | 0.45 | 5.77 | <0.001 | 1.70-3.45 |
| Anticipation (No vs. Yes) | | 1.36 | 0.45 | 3.03 | 0.002 | 0.48-2.25 | -0.33 | 0.46 | -0.72 | 0.473 | -1.23-0.57 |
| Approach Velocity (Covariate) | |  |  |  |  |  | -5.21 | 0.47 | -11.17 | <0.001 | -6.12- -4.30 |
| Interaction barefoot/minimalist x Anticipation | | 0.30 | 0.64 | 0.47 | 0.639 | ‚-0.95-1.55 | 0.21 | 0.62 | 0.34 | 0.738 | ‚-1.00-1.42 |
| Interaction standard/minimalist x Anticipation | | -0.48 | 0.63 | -0.77 | 0.443 | ‚-1.72-0.75 | -0.66 | 0.61 | -1.08 | 0.282 | ‚-1.86-0.54 |
| **Ground contact time [s]** | |  |  |  |  |  |  |  |  |  |  |
| (Intercept) | | 0.33 | 0.01 | 40.67 | <0.001 | 0.31-0.34 | 0.36 | 0.01 | 25.62 | <0.001 | 0.33-0.39 |
| Barefoot vs. minimalist | | -0.01 | 0.003 | -2.36 | 0.019 | -0.01- -0.001 | -0.01 | 0.004 | -2.83 | 0.005 | -0.02- -0.003 |
| Standard vs. minimalist | | -0.001 | 0.003 | -0.39 | 0.699 | -0.008-0.005 | 0.0001 | 0.004 | 0.04 | 0.967 | -0.007- 0.007 |
| Anticipation (No vs. Yes) | | 0.01 | 0.003 | 1.65 | 0.0998 | -0.001-0.01 | 0.002 | 0.004 | 0.56 | 0.573 | -0.005- 0.009 |
| Approach Velocity (Covariate) | |  |  |  |  |  | -0.01 | 0.004 | -2.98 | 0.003 | -0.02- -0.004 |
| Interaction barefoot/minimalist x Anticipation | | 0.003 | 0.01 | 0.71 | 0.477 | ‚-0.01-0.01 | 0.003 | 0.005 | 0.67 | 0.501 | ‚-0.01-0.01 |
| Interaction standard/minimalist x Anticipation | | -0.004 | 0.01 | -0.82 | 0.412 | ‚-0.01-0.01 | -0.004 | 0.005 | -0.90 | 0.368 | ‚-0.01-0.01 |
| **Vertical instantaneous load [BW*s-1]** | | |  |  |  |  |  |  |  |  |  |
| (Intercept) | | 229.91 | 8.14 | 28.25 | <0.001 | 213.89-245.95 | 56.67 | 20.05 | 2.83 | 0.005 | 17.56-95.87 |
| Barefoot vs. minimalist | | -1.76 | 5.71 | -0.31 | 0.758 | -12.94-9.42 | 6.86 | 5.66 | 1.21 | 0.226 | -4.23-17.94 |
| Standard vs. minimalist | | -80.13 | 5.70 | -14.05 | <0.001 | -91.3- -68.97 | -87.64 | 5.63 | -15.56 | <0.001 | -98.67- -76.61 |
| Anticipation (No vs. Yes) | | -17.52 | 5.62 | -3.12 | 0.002 | -28.53- -6.51 | 0.28 | 5.82 | 0.05 | 0.961 | -11.10-11.67 |
| Approach Velocity (Covariate) | |  |  |  |  |  | 54.93 | 5.86 | 9.37 | <0.001 | 43.46-66.41 |
| Interaction barefoot/minimalist x Anticipation | | --5.34 | 7.96 | -0.67 | 0.502 | ‚-20.94-10.25 | -4.29 | 7.79 | -0.55 | 0.582 | ‚-19.54-10.96 |
| Interaction standard/minimalist x Anticipation | | 15.99 | 7.90 | 2.02 | 0.043 | 0.51-31.46 | 17.91 | 7.73 | 2.32 | 0.021 | 2.77-33.05 |
| **Vertical average load [BW*s-1]** |  | |  |  |  |  |  |  |  |  |  |
| (Intercept) | | 52.56 | 3.48 | 15.10 | <0.001 | 45.69-59.44 | -5.88 | 7.05 | -0.83 | 0.406 | -19.66-8.00 |
| Barefoot vs. minimalist | | -6.64 | 1.89 | -3.51 | <0.001 | -10.34- -2.94 | -3.72 | 1.87 | -1.97 | 0.047 | -7.39- -0.05 |
| Standard vs. minimalist | | 8.64 | 1.89 | 4.58 | <0.001 | 4.94-12.33 | 6.11 | 1.86 | 3.28 | 0.001 | 2.46-9.76 |
| Anticipation (No vs. Yes) | | -7.56 | 1.86 | -4.07 | <0.001 | -11.21- -3.92 | -1.56 | 1.93 | -0.81 | 0.417 | -5.33-2.21 |
| Approach Velocity (Covariate) | |  |  |  |  |  | 18.53 | 1.97 | 9.43 | <0.001 | 14.68-22.38 |
| Interaction barefoot/minimalist x Anticipation | | -0.20 | 2.64 | 0.08 | 0.950 | -4.96-5.36 | 0.55 | 2.58 | 0.21 | 0.830 | -4.49-5.60 |
| Interaction standard/minimalist x Anticipation | | 0.08 | 2.62 | 0.03 | 0.975 | -5.04-5.21 | 0.73 | 2.56 | 0.29 | 0.773 | -4.27-5.74 |
| **Mean dynamic friction coefficient** | | |  |  |  |  |  |  |  |  |  |
| (Intercept) | | 0.56 | 0.01 | 98.2 | <0.001 | 0.54-0.57 | 0.42 | 0.01 | 33.83 | <0.001 | 0.40-0.45 |
| Barefoot vs. minimalist | | -0.05 | 0.004 | -15.41 | <0.001 | -0.06- -0.05 | -0.05 | 0.003 | -13.76 | <0.001 | -0.05- -0.04 |
| Standard vs. minimalist | | 0.02 | 0.004 | 5.70 | <0.001 | 0.01-0.03 | 0.01 | 0.003 | 4.18 | <0.001 | 0.008-0.02 |
| Anticipation (No vs. Yes) | | -0.002 | 0.003 | -0.69 | 0.492 | -0.01-0.004 | 0.01 | 0.004 | 3.19 | 0.001 | 0.004-0.02 |
| Approach Velocity (Covariate) | |  |  |  |  |  | 0.04 | 0.004 | 11.76 | <0.001 | 0.04-0.05 |
| Interaction barefoot/minimalist x Anticipation | | 0.01 | 0.005 | 1.45 | 0.147 | ‚-0.003-0.02 | 0.01 | 0.005 | 1.66 | 0.097 | ‚-0.001-0.02 |
| Interaction standard/minimalist x Anticipation | | 0.01 | 0.005 | 1.90 | 0.057 | ‚-0.0003-0.02 | 0.01 | 0.005 | 2.27 | 0.023 | 0.001-0.02 |
| **Mean force in sideward direction [BW]** | | |  |  |  |  |  |  |  |  |  |
| (Intercept) | | -0.52 | 0.01 | -57.89 | <0.001 | -0.54- -0.51 | -0.47 | 0.02 | -22.62 | <0.001 | -0.51- -0.43 |
| Barefoot vs. minimalist | | 0.05 | 0.006 | 8.56 | <0.001 | 0.04-0.06 | 0.05 | 0.006 | 7.99 | <0.001 | 0.03-0.06 |
| Standard vs. minimalist | | -0.03 | 0.006 | -5.97 | <0.001 | -0.04- -0.02 | -0.03 | 0.006 | -5.53 | <0.001 | -0.04- -0.02 |
| Anticipation (No vs. Yes) | | -0.03 | 0.006 | -5.78 | <0.001 | -0.04- -0.02 | -0.04 | 0.006 | -6.39 | <0.001 | -0.05- -0.03 |
| Approach Velocity (Covariate) | |  |  |  |  |  | -0.02 | 0.006 | -2.80 | 0.005 | -0.03- -0.005 |
| Interaction barefoot/minimalist x Anticipation | | -0.003 | 0.008 | -0.43 | 0.668 | ‚-0.02-0.01 | -0.004 | 0.008 | -0.47 | 0.641 | ‚-0.02-0.01 |
| Interaction standard/minimalist x Anticipation | | -0.01 | 0.008 | -1.08 | 0.281 | ‚-0.02-0.01 | -0.01 | 0.008 | -1.15 | 0.249 | ‚-0.02-0.01 |
| **Mean force in forward direction [BW]** | | |  |  |  |  |  |  |  |  |  |
| (Intercept) | | -0.47 | 0.009 | -54.81 | <0.001 | -0.49- -0.45 | -0.19 | 0.02 | -10.75 | <0.001 | -0.22- -0.16 |
| Barefoot vs. minimalist | | 0.06 | 0.005 | 12.23 | <0.001 | 0.05-0.07 | 0.05 | 0.005 | 10.09 | <0.001 | 0.04-0.06 |
| Standard vs. minimalist | | -0.03 | 0.005 | -5.91 | <0.001 | -0.04- -0.02 | -0.02 | 0.005 | -3.85 | <0.001 | -0.03- -0.01 |
| Anticipation (No vs. Yes) | | 0.05 | 0.005 | 9.37 | <0.001 | 0.04-0.06 | 0.02 | 0.005 | 3.84 | <0.001 | 0.01-0.03 |
| Approach Velocity (Covariate) | |  |  |  |  |  | -0.09 | 0.005 | -17.23 | <0.001 | -0.10- -0.08 |
| Interaction barefoot/minimalist x Anticipation | | -0.01 | 0.007 | -1.88 | 0.060 | ‚-0.03-0.001 | -0.02 | 0.007 | -2.25 | 0.025 | ‚-0.03- -0.002 |
| Interaction standard/minimalist x Anticipation | | -0.004 | 0.007 | -0.57 | 0.567 | ‚-0.02-0.01 | -0.01 | 0.007 | -1.06 | 0.292 | ‚-0.02-0.01 |
| **Mean force in vertical direction [BW]** | | |  |  |  |  |  |  |  |  |  |
| (Intercept) | | 1.39 | 0.02 | 91.17 | <0.001 | 1.36-1.42 | 1.23 | 0.03 | 40.49 | <0.001 | 1.17-1.29 |
| Barefoot vs. minimalist | | -0.03 | 0.01 | -3.62 | <0.001 | -0.04- -0.01 | -0.02 | 0.01 | -2.59 | 0.01 | -0.04- -0.005 |
| Standard vs. minimalist | | 0.01 | 0.01 | 1.28 | 0.20 | -0.005-0.03 | 0.003 | 0.01 | 0.41 | 0.685 | -0.01-0.02 |
| Anticipation (No vs. Yes) | | -0.004 | 0.01 | -0.49 | 0.626 | -0.02-0.01 | 0.01 | 0.01 | 1.56 | 0.119 | -0.003-0.03 |
| Approach Velocity (Covariate) | |  |  |  |  |  | 0.05 | 0.01 | 6.09 | <0.001 | 0.03-0.07 |
| Interaction barefoot/minimalist x Anticipation | | -0.01 | 0.01 | -0.74 | 0.459 | ‚-0.03-0.01 | -0.01 | 0.01 | -0.67 | 0.506 | ‚-0.03-0.01 |
| Interaction standard/minimalist x Anticipation | | -0.002 | 0.01 | -0.18 | 0.857 | ‚-0.02-0.02 | -0.0003 | 0.01 | -0.02 | 0.981 | ‚-0.02-0.02 |
| **Maximum braking force in sideward direction [BW]** | | | |  |  |  |  |  |  |  |  |
| (Intercept) | | -0.70 | 0.02 | -38.38 | <0.001 | -0.73- -0.65 | -0.45 | 0.04 | -11.65 | <0.001 | -0.52- -0.37 |
| Barefoot vs. minimalist | | 0.08 | 0.01 | 7.62 | <0.001 | 0.06-0.10 | 0.07 | 0.01 | 6.44 | <0.001 | 0.05-0.09 |
| Standard vs. minimalist | | -0.11 | 0.01 | -10.22 | <0.001 | -0.13- -0.09 | -0.10 | 0.01 | -9.25 | <0.001 | -0.12- -0.08 |
| Anticipation (No vs. Yes) | | -0.04 | 0.01 | -3.85 | <0.001 | -0.06- -0.02 | -6.42 | 0.01 | -5.99 | <0.001 | -0.09- -0.04 |
| Approach Velocity (Covariate) | |  |  |  |  |  | -0.08 | 0.01 | -7.01 | <0.001 | -0.10- -0.06 |
| Interaction barefoot/minimalist x Anticipation | | 0.002 | 0.01 | 0.15 | 0.881 | ‚-0.03-0.03 | 0.001 | 0.01 | 0.06 | 0.955 | ‚-0.03-0.03 |
| Interaction standard/minimalist x Anticipation | | -0.02 | 0.01 | -1.04 | 0.297 | ‚-0.04-0.01 | -0.02 | 0.01 | -1.24 | 0.216 | ‚-0.05-0.01 |
| **Maximum braking force in forward direction [BW]** | | | |  |  |  |  |  |  |  |  |
| (Intercept) | | -1.01 | 0.03 | -39.58 | <0.001 | -0.11- -0.96 | 0.21 | 0.05 | 3.96 | <0.001 | 0.10-0.31 |
| Barefoot vs. minimalist | | 0.19 | 0.02 | 10.72 | <0.001 | 0.15-0.21 | 0.12 | 0.01 | 8.07 | <0.001 | 0.09-0.15 |
| Standard vs. minimalist | | -0.16 | 0.02 | -9.47 | <0.001 | -0.19- -0.13 | -0.11 | 0.01 | -7.29 | <0.001 | -0.14- -0.08 |
| Anticipation (No vs. Yes) | | 0.12 | 0.02 | 7.13 | <0.001 | 0.09-0.15 | -0.01 | 0.02 | -0.48 | 0.630 | -0.04-0.02 |
| Approach Velocity (Covariate) | |  |  |  |  |  | -0.39 | 0.02 | -25.27 | <0.001 | -0.42- -0.36 |
| Interaction barefoot/minimalist x Anticipation | | -0.04 | 0.02 | -1.77 | 0.077 | ‚-0.09-0.004 | -0.05 | 0.02 | -2.38 | 0.017 | ‚-0.09- -0.01 |
| Interaction standard/minimalist x Anticipation | | 0.06 | 0.02 | 2.66 | 0.008 | 0.02-0.11 | 0.05 | 0.02 | 2.43 | 0.015 | 0.01-0.09 |
| **Maximum braking force in vertical direction [BW]** | | | |  |  |  |  |  |  |  |  |
| (Intercept) | | 2.30 | 0.04 | 52.70 | <0.001 | 2.22-2.39 | 0.78 | 0.10 | 8.18 | <0.001 | 0.60-0.97 |
| Barefoot vs. minimalist | | -0.18 | 0.03 | -6.48 | <0.001 | -0.02- -0.13 | -0.11 | 0.03 | -4.00 | <0.001 | -0.16- -0.05 |
| Standard vs. minimalist | | 0.07 | 0.03 | 2.50 | 0.013 | 0.02-0.13 | 0.01 | 0.03 | 0.19 | 0.846 | -0.05-0.06 |
| Anticipation (No vs. Yes) | | -0.06 | 0.03 | -2.28 | 0.023 | -0.12- -0.01 | 0.09 | 0.03 | 3.46 | <0.001 | 0.04-0.15 |
| Approach Velocity (Covariate) | |  |  |  |  |  | 0.48 | 0.03 | 17.52 | <0.001 | 0.43-0.54 |
| Interaction barefoot/minimalist x Anticipation | | -0.02 | 0.04 | -0.45 | 0.655 | ‚-0.09-0.06 | -0.01 | 0.04 | -0.25 | 0.804 | ‚-0.08-0.06 |
| Interaction standard/minimalist x Anticipation | | -0.01 | 0.04 | -0.25 | 0.802 | ‚-0.09-0.07 | 0.01 | 0.04 | 0.18 | 0.858 | ‚-0.06-0.08 |
| **Maximum propulsion force in sideward direction [BW]** | | | |  |  |  |  |  |  |  |  |
| (Intercept) | | -0.80 | 0.01 | -59.72 | <0.001 | -0.83- -0.77 | -0.75 | 0.03 | -23.92 | <0.001 | -0.81- -0.69 |
| Barefoot vs. minimalist | | 0.06 | 0.01 | 6.52 | <0.001 | 0.04-0.07 | 0.05 | 0.01 | 6.12 | <0.001 | 0.04-0.07 |
| Standard vs. minimalist | | -0.03 | 0.01 | -3.78 | <0.001 | -0.05- -0.02 | -0.03 | 0.01 | -3.48 | <0.001 | -0.05- -0.01 |
| Anticipation (No vs. Yes) | | -0.03 | 0.01 | -3.59 | <0.001 | -0.05- -0.01 | -0.04 | 0.01 | -4.01 | <0.001 | -0.05- -0.02 |
| Approach Velocity (Covariate) | |  |  |  |  |  | -0.02 | 0.01 | -1.88 | 0.061 | -0.03-0.001 |
| Interaction barefoot/minimalist x Anticipation | | -0.006 | 0.01 | -0.47 | 0.636 | ‚-0.03-0.02 | -0.01 | 0.01 | -0.50 | 0.618 | ‚-0.03-0.02 |
| Interaction standard/minimalist x Anticipation | | -0.01 | 0.01 | -1.15 | 0.251 | ‚-0.04-0.01 | -0.01 | 0.01 | -1.20 | 0.231 | ‚-0.04-0.01 |
| **Maximum propulsion force in forward direction [BW]** | | | |  |  |  |  |  |  |  |  |
| (Intercept) | | -0.67 | 0.02 | -36.63 | <0.001 | -0.70- -0.63 | -0.30 | 0.04 | -8.06 | <0.001 | -0.37- -0.23 |
| Barefoot vs. minimalist | | 0.06 | 0.01 | 5.44 | <0.001 | 0.04-0.08 | 0.04 | 0.01 | 3.68 | <0.001 | 0.02-0.06 |
| Standard vs. minimalist | | -0.02 | 0.01 | -1.73 | 0.084 | -0.04-0.002 | -0.002 | 0.01 | -0.20 | 0.842 | -0.02-0.02 |
| Anticipation (No vs. Yes) | | 0.07 | 0.01 | 6.49 | <0.001 | 0.05-0.09 | 0.03 | 0.01 | 2.65 | 0.008 | 0.01-0.05 |
| Approach Velocity (Covariate) | |  |  |  |  |  | -0.12 | 0.01 | -11.06 | <0.001 | -0.14- -0.10 |
| Interaction barefoot/minimalist x Anticipation | | 0.001 | 0.01 | 0.10 | 0.923 | ‚-0.03-0.03 | -0.001 | 0.01 | -0.05 | 0.961 | ‚-0.03-0.03 |
| Interaction standard/minimalist x Anticipation | | -0.01 | 0.01 | -1.02 | 0.309 | ‚-0.04-0.01 | -0.02 | 0.01 | -1.33 | 0.182 | ‚-0.05-0.01 |
| **Maximum propulsion force in vertical direction [BW]** | | | |  |  |  |  |  |  |  |  |
| (Intercept) | | 1.80 | 0.03 | 59.99 | <0.001 | 1.74-1.86 | 1.76 | 0.06 | 30.68 | <0.001 | 1.65-1.87 |
| Barefoot vs. minimalist | | -0.01 | 0.01 | -0.80 | 0.426 | -0.04-0.02 | -0.01 | 0.01 | -0.66 | 0.511 | -0.04-0.02 |
| Standard vs. minimalist | | -0.001 | 0.01 | -0.09 | 0.927 | -0.03-0.03 | -0.003 | 0.01 | -0.20 | 0.842 | -0.03-0.03 |
| Anticipation (No vs. Yes) | | -0.003 | 0.01 | -0.23 | 0.819 | -0.03-0.02 | 0.0006 | 0.02 | 0.04 | 0.971 | -0.03-0.03 |
| Approach Velocity (Covariate) | |  |  |  |  |  | 0.01 | 0.02 | 0.76 | 0.448 | -0.02-0.04 |
| Interaction barefoot/minimalist x Anticipation | | -0.02 | 0.02 | -0.84 | 0.401 | ‚-0.06-0.02 | -0.02 | 0.02 | -0.83 | 0.407 | ‚-0.06-0.02 |
| Interaction standard/minimalist x Anticipation | | 0.02 | 0.02 | 0.77 | 0.440 | ‚-0.02-0.05 | 0.02 | 0.02 | 0.79 | 0.429 | ‚-0.02-0.06 |
| **Braking time [s]** | |  |  |  |  |  |  |  |  |  |  |
| (Intercept) | | 0.14 | 0.004 | 32.70 | <0.001 | 0.13-0.15 | 0.16 | 0.01 | 16.23 | <0.001 | 0.14-0.18 |
| Barefoot vs. minimalist | | -0.01 | 0.003 | -3.65 | <0.001 | -0.01- -0.004 | -0.01 | 0.003 | -3.88 | <0.001 | -0.02- -0.01 |
| Standard vs. minimalist | | 0.01 | 0.003 | 2.45 | 0.015 | 0.001-0.01 | 0.01 | 0.003 | 2.66 | 0.008 | 0.002-0.01 |
| Anticipation (No vs. Yes) | | -0.001 | 0.003 | -0.38 | 0.703 | -0.006-0.004 | -0.002 | 0.003 | -0.92 | 0.359 | -0.01-0.003 |
| Approach Velocity (Covariate) | |  |  |  |  |  | -0.01 | 0.003 | -1.68 | 0.093 | -0.01-0.001 |
| Interaction barefoot/minimalist x Anticipation | | 0.01 | 0.004 | 1.56 | 0.119 | ‚-0.001-0.01 | 0.006 | 0.004 | 1.54 | 0.125 | ‚-0.002-0.01 |
| Interaction standard/minimalist x Anticipation | | -0.004 | 0.004 | -1.08 | 0.280 | ‚-0.01-0.003 | -0.004 | 0.004 | -1.13 | 0.261 | ‚-0.01-0.003 |
| **Propulsion time [s]** | |  |  |  |  |  |  |  |  |  |  |
| (Intercept) | | 0.18 | 0.004 | 42.62 | <0.001 | 0.18-0.19 | 0.21 | 0.01 | 23.57 | <0.001 | 0.19-0.22 |
| Barefoot vs. minimalist | | 0.001 | 0.002 | 0.55 | 0.584 | -0.003-0.006 | 0.0001 | 0.002 | 0.07 | 0.948 | -0.004-0.005 |
| Standard vs. minimalist | | -0.008 | 0.002 | -3.40 | <0.001 | -0.002- -0.003 | -0.01 | 0.002 | -2.97 | 0.003 | -0.01- -0.002 |
| Anticipation (No vs. Yes) | | 0.007 | 0.002 | 2.95 | 0.003 | 0.002-0.01 | 0.004 | 0.002 | 1.84 | 0.07 | -0.0003-0.009 |
| Approach Velocity (Covariate) | |  |  |  |  |  | -0.01 | 0.002 | -2.85 | 0.004 | -0.01- -0.002 |
| Interaction barefoot/minimalist x Anticipation | | -0.002 | 0.003 | -0.70 | 0.486 | ‚-0.01-0.004 | -0.002 | 0.003 | -0.74 | 0.461 | ‚-0.01-0.004 |
| Interaction standard/minimalist x Anticipation | | -0.00005 | 0.003 | -0.02 | 0.988 | ‚-0.01-0.01 | -0.0003 | 0.003 | -0.09 | 0.929 | ‚-0.006-0.006 |
| **Ankle joint angle [°]** | |  |  |  |  |  |  |  |  |  |  |
| (Intercept) | | -18.37 | 0.99 | -18.50 | <0.001 | -20.33- -16.41 | -13.7 | 2.05 | -6.68 | <0.001 | -17.71- -9.69 |
| Barefoot vs. minimalist | | 0.06 | 0.53 | 0.12 | 0.905 | -0.98-1.11 | -0.17 | 0.54 | -0.32 | 0.752 | -1.23-0.89 |
| Standard vs. minimalist | | 7.50 | 0.53 | 14.06 | <0.001 | 6.46-8.55 | 7.70 | 0.54 | 14.31 | <0.001 | 6.65-8.76 |
| Anticipation (No vs. Yes) | | 1.63 | 0.53 | 3.11 | 0.002 | 0.60-2.66 | 1.15 | 0.56 | 2.07 | 0.039 | 0.06-2.24 |
| Approach Velocity (Covariate) | |  |  |  |  |  | -1.48 | 0.57 | -2.61 | 0.009 | -2.59- -0.37 |
| Interaction barefoot/minimalist x Anticipation | | -0.02 | 0.75 | -0.03 | 0.980 | ‚-1.48-1.44 | -0.05 | 0.74 | -0.06 | 0.952 | ‚-1.50-1.41 |
| Interaction standard/minimalist x Anticipation | | -1.74 | 0.74 | -2.35 | 0.019 | ‚-3.18- -0.29 | -1.79 | 0.74 | -2.42 | 0.016 | ‚-3.23- -0.34 |
| **Ankle joint range of motion [°]** | |  |  |  |  |  |  |  |  |  |  |
| (Intercept) | | 43.67 | 0.77 | 56.87 | <0.001 | 42.16-45.19 | 44.36 | 1.49 | 29.72 | <0.001 | 41.44-47.28 |
| Barefoot vs. minimalist | | -0.32 | 0.38 | -0.84 | 0.404 | -1.06-0.43 | -0.35 | 0.39 | -0.91 | 0.361 | -1.11-0.40 |
| Standard vs. minimalist | | 0.36 | 0.38 | 0.95 | 0.343 | -0.38-1.10 | 0.39 | 0.38 | 1.02 | 0.310 | -0.36-1.14 |
| Anticipation (No vs. Yes) | | 0.92 | 0.37 | 2.47 | 0.014 | 0.19-1.66 | 0.85 | 0.40 | 2.15 | 0.032 | 0.08-1.63 |
| Approach Velocity (Covariate) | |  |  |  |  |  | -0.22 | 0.41 | -0.54 | 0.592 | -1.01-0.58 |
| Interaction barefoot/minimalist x Anticipation | | -0.98 | 0.53 | -1.84 | 0.066 | ‚-2.02-0.06 | -0.98 | 0.53 | -1.85 | 0.065 | ‚-2.02-0.06 |
| Interaction standard/minimalist x Anticipation | | -0.68 | 0.53 | -1.28 | 0.200 | ‚-1.71-0.36 | -0.68 | 0.53 | -1.30 | 0.195 | ‚-1.71-0.35 |
| **Knee joint angle [°]** | |  |  |  |  |  |  |  |  |  |  |
| (Intercept) | | -23.11 | 0.73 | -31.67 | <0.001 | -24.55- -21.67 | -36.75 | 1.68 | -21.81 | <0.001 | -40.05- -33.45 |
| Barefoot vs. minimalist | | -0.95 | 0.47 | -2.05 | 0.041 | -1.87- -0.04 | -0.27 | 0.46 | -0.58 | 0.562 | -1.17-0.64 |
| Standard vs. minimalist | | 0.71 | 0.46 | 1.53 | 0.125 | -0.20-1.62 | 0.13 | 0.46 | 0.28 | 0.779 | -0.77-1.03 |
| Anticipation (No vs. Yes) | | -3.79 | 0.46 | -8.27 | <0.001 | 4.69- -2.89 | -2.38 | 0.48 | -5.01 | <0.001 | -3.31- -1.45 |
| Approach Velocity (Covariate) | |  |  |  |  |  | 4.32 | 0.48 | 8.97 | <0.001 | 3.38-5.27 |
| Interaction barefoot/minimalist x Anticipation | | 1.67 | 0.65 | 2.58 | 0.010 | 0.40-2.94 | 1.75 | 0.64 | 2.75 | 0.006 | 0.50-2.99 |
| Interaction standard/minimalist x Anticipation | | 0.57 | 0.64 | 0.88 | 0.377 | ‚-0.69-1.83 | 0.72 | 0.63 | 1.13 | 0.257 | ‚-0.52-1.95 |
| **Knee joint range of motion [°]** | |  |  |  |  |  |  |  |  |  |  |
| (Intercept) | | 42.67 | 1.10 | 38.67 | <0.001 | 40.49-44.85 | 45.57 | 1.99 | 22.92 | <0.001 | 41.68-49.46 |
| Barefoot vs. minimalist | | -2.42 | 0.49 | -4.92 | <0.001 | -3.38- -1.46 | -2.57 | 0.50 | -5.15 | <0.001 | -3.54- -1.59 |
| Standard vs. minimalist | | 0.26 | 0.49 | 0.53 | 0.594 | -0.70-1.22 | 0.39 | 0.50 | 0.78 | 0.436 | -0.58-1.36 |
| Anticipation (No vs. Yes) | | 0.86 | 0.48 | 1.77 | 0.076 | -0.09-1.81 | 0.56 | 0.51 | 1.09 | 0.276 | -0.45-1.56 |
| Approach Velocity (Covariate) | |  |  |  |  |  | -0.92 | 0.53 | -1.75 | 0.080 | -1.95-0.11 |
| Interaction barefoot/minimalist x Anticipation | | 1.07 | 0.69 | 1.56 | 0.119 | ‚-0.27-2.41 | 1.05 | 0.69 | 1.54 | 0.125 | ‚-0.29-2.39 |
| Interaction standard/minimalist x Anticipation | | ‚-0.46 | 0.68 | -0.68 | 0.498 | ‚-1.79-0.87 | -0.49 | 0.68 | -0.72 | 0.470 | ‚-1.82-0.84 |
| **Peak muscle activity of the gastrocnemius lateralis [uV]** | | | |  |  |  |  |  |  |  |  |
| (Intercept) | | 0.0004 | <0.001 | 13.21 | <0.001 | 0.0004-0.0005 | 0.001 | <0.001 | 8.19 | <0.001 | 0.0003-0.0006 |
| Barefoot vs. minimalist | | <-0.001 | <0.001 | -3.51 | <0.001 | -0.00007- -0.00002 | -0.0001 | <0.001 | -3.52 | <0.001 | -0.00007- -0.00002 |
| Standard vs. minimalist | | <0.001 | <0.001 | 0.339 | 0.734 | -0.00002-0.00003 | <0.001 | <0.001 | 0.39 | 0.698 | -0.00002- -0.00003 |
| Anticipation (No vs. Yes) | | <0.001 | <0.001 | 0.262 | 0.793 | -0.00002-0.00003 | <0.001 | <0.001 | 0.13 | 0.900 | -0.00003-0.00003 |
| Approach Velocity (Covariate) | |  |  |  |  |  | <-0.001 | <0.001 | -0.36 | 0.716 | -0.00003-0.00002 |
| Interaction barefoot/minimalist x Anticipation | | <0.001 | <0.001 | 0.04 | 0.972 | ‚-0.00004-0.00004 | <0.001 | <0.001 | 0.03 | 0.975 | ‚-0.00004-0.00004 |
| Interaction standard/minimalist x Anticipation | | <0.001 | <0.001 | 1.03 | 0.305 | ‚-0.00002-0.00005 | <0.001 | <0.001 | 1.02 | 0.310 | ‚-0.00002-0.00005 |
| **Peak muscle activity of the gastrocnemius medialis [uV]** | | | |  |  |  |  |  |  |  |  |
| (Intercept) | | 0.0004 | <0.001 | 14.06 | <0.001 | 0.0003-0.0005 | 0.0003 | <0.001 | 6.63 | <0.001 | 0.0002-0.0004 |
| Barefoot vs. minimalist | | <-0.001 | <0.001 | -0.72 | 0.470 | -0.00003-0.00001 | <-0.001 | <0.001 | -0.28 | 0.782 | -0.00002-0.00002 |
| Standard vs. minimalist | | <-0.001 | <0.001 | -0.20 | 0.839 | -0.00002-0.00002 | <-0.001 | <0.001 | -0.57 | 0.567 | -0.00003-0.00001 |
| Anticipation (No vs. Yes) | | <0.001 | <0.001 | 1.27 | 0.205 | -0.000007-0.00003 | <0.001 | <0.001 | 2.06 | 0.039 | 0.000001-0.00005 |
| Approach Velocity (Covariate) | |  |  |  |  |  | <0.001 | <0.001 | 2.59 | 0.010 | 0.000007-0.00005 |
| Interaction barefoot/minimalist x Anticipation | | <-0.001 | <0.001 | -2.38 | 0.018 | ‚-0.00007- -0.000006 | <-0.001 | <0.001 | -2.35 | 0.019 | ‚-0.00006- -0.000006 |
| Interaction standard/minimalist x Anticipation | | <-0.001 | <0.001 | -0.09 | 0.928 | ‚-0.00003-0.00003 | <-0.001 | <0.001 | -0.02 | 0.982 | ‚-0.00003-0.00003 |
| **Peak muscle activity of the tibialis anterior [uV]** | | | |  |  |  |  |  |  |  |  |
| (Intercept) | | 0.0003 | <0.001 | 18.96 | <0.001 | 0.0003-0.0004 | 0.0002 | <0.001 | 5.13 | <0.001 | 0.0001-0.0002 |
| Barefoot vs. minimalist | | <0.001 | <0.001 | 0.58 | 0.564 | -0.00001-0.00002 | <0.001 | <0.001 | 1.48 | 0.138 | -0.000004-0.00003 |
| Standard vs. minimalist | | <0.001 | <0.001 | 0.14 | 0.892 | -0.00002-0.00002 | <-0.001 | <0.001 | -0.64 | 0.521 | -0.00002-0.00001 |
| Anticipation (No vs. Yes) | | <-0.001 | <0.001 | -4.32 | <0.001 | -0.00005- -0.00002 | <-0.001 | <0.001 | -2.30 | 0.022 | -0.00004- -0.000003 |
| Approach Velocity (Covariate) | |  |  |  |  |  | <0.001 | <0.001 | 5.45 | <0.001 | 0.00003-0.00007 |
| Interaction barefoot/minimalist x Anticipation | | <0.001 | <0.001 | 0.68 | 0.499 | ‚-0.00002-0.00003 | <0.001 | <0.001 | 0.75 | 0.451 | ‚-0.00001-0.00003 |
| Interaction standard/minimalist x Anticipation | | <0.001 | <0.001 | 0.24 | 0.809 | ‚-0.00002-0.00003 | <0.001 | <0.001 | 0.39 | 0.700 | ‚-0.00002-0.00003 |
| **Mean muscle activity of the gastrocnemius lateralis [uV]** | | | |  |  |  |  |  |  |  |  |
| (Intercept) | | 0.0002 | <0.001 | 14.53 | <0.001 | 0.0001-0.0002 | 0.0002 | <0.001 | 8.37 | <0.001 | 0.0001-0.0002 |
| Barefoot vs. minimalist | | <-0.001 | <0.001 | -5.75 | <0.001 | -0.00003- -0.00002 | <-0.001 | <0.001 | -5.51 | <0.001 | -0.00003- -0.00002 |
| Standard vs. minimalist | | <-0.001 | <0.001 | -0.59 | 0.558 | -0.00001-0.000006 | <-0.001 | <0.001 | -0.71 | 0.476 | -0.00001-0.000005 |
| Anticipation (No vs. Yes) | | <0.001 | <0.001 | 2.74 | 0.006 | 0.000003-0.00002 | <0.001 | <0.001 | 2.89 | 0.004 | 0.000004-0.00002 |
| Approach Velocity (Covariate) | |  |  |  |  |  | <0.001 | <0.001 | 0.93 | 0.351 | -0.000005-0.00001 |
| Interaction barefoot/minimalist x Anticipation | | <-0.001 | <0.001 | -1.25 | 0.213 | ‚-0.00002-0.000004 | <-0.001 | <0.001 | -1.23 | 0.217 | ‚-0.00002-0.000004 |
| Interaction standard/minimalist x Anticipation | | <0.001 | <0.001 | 0.99 | 0.324 | ‚-0.000006-0.00002 | <0.001 | <0.001 | 1.01 | 0.312 | ‚-0.000006-0.00002 |
| **Mean muscle activity of gastrocnemius medialis [uV]** | | | |  |  |  |  |  |  |  |  |
| (Intercept) | | 0.0002 | <0.001 | 14.54 | <0.001 | 0.0002-0.0002 | 0.0001 | <0.001 | 6.92 | <0.001 | 0.00009-0.0002 |
| Barefoot vs. minimalist | | -0.00001 | <0.001 | -3.78 | <0.001 | -0.00002- -0.000007 | <-0.001 | <0.001 | -3.04 | 0.002 | -0.00002- -0.000004 |
| Standard vs. minimalist | | -0.00002 | <0.001 | -3.95 | <0.001 | -0.00002- -0.000008 | <-0.001 | <0.001 | -4.52 | <0.001 | -0.00002- -0.00001 |
| Anticipation (No vs. Yes) | | <0.001 | <0.001 | 0.35 | 0.728 | -0.000006-0.000009 | <0.001 | <0.001 | 1.73 | 0.084 | -0.000001-0.00001 |
| Approach Velocity (Covariate) | |  |  |  |  |  | <0.001 | <0.001 | 4.19 | <0.001 | 0.000009-0.00003 |
| Interaction barefoot/minimalist x Anticipation | | <-0.001 | <0.001 | -1.45 | 0.148 | ‚-0.00002-0.000003 | <-0.001 | <0.001 | -1.40 | 0.163 | ‚-0.00002-0.000003 |
| Interaction standard/minimalist x Anticipation | | <0.001 | <0.001 | 1.25 | 0.212 | ‚-0.000004-0.00002 | <0.001 | <0.001 | 1.37 | 0.172 | ‚-0.000003-0.00002 |
| **Mean muscle activity of the tibialis anterior [uV]** | | | |  |  |  |  |  |  |  |  |
| (Intercept) | | 0.0002 | <0.001 | 17.25 | <0.001 | 0.0001-0.0002 | 0.00006 | 0.00001 | 4.24 | <0.001 | 0.00003-0.00009 |
| Barefoot vs. minimalist | | 0.00001 | <0.001 | 2.89 | 0.004 | 0.000003-0.00002 | <0.001 | <0.001 | 4.21 | <0.001 | 0.000008-0.00002 |
| Standard vs. minimalist | | <0.001 | <0.001 | 1.47 | 0.141 | -0.000002-0.00001 | <0.001 | <0.001 | 0.36 | 0.720 | -0.000006-0.000008 |
| Anticipation (No vs. Yes) | | <-0.001 | <0.001 | -4.24 | <0.001 | -0.00002- -0.000008 | <-0.001 | <0.001 | -1.45 | 0.148 | -0.00001-0.000002 |
| Approach Velocity (Covariate) | |  |  |  |  |  | <0.001 | <0.001 | 7.83 | <0.001 | 0.00002-0.00004 |
| Interaction barefoot/minimalist x Anticipation | | <-0.001 | <0.001 | -0.24 | 0.811 | ‚-0.00001-0.000008 | <-0.001 | <0.001 | -0.14 | 0.891 | ‚-0.00001-0.000009 |
| Interaction standard/minimalist x Anticipation | | <-0.001 | <0.001 | -0.52 | 0.602 | ‚-0.00001-0.000007 | <-0.001 | <0.001 | -0.33 | 0.745 | ‚-0.00001-0.000008 |
| **Muscle activity of gastrocnemius lateralis at initial ground contact [uV]** | |  |  |  |  |  |  |  |  |  |  |
| (Intercept) | | 0.0002 | 0.00002 | 11.48 | <0.001 | 0.0001-0.0002 | 0.0001 | <0.001 | 3.12 | 0.002 | 0.00004-0.0002 |
| Barefoot vs. minimalist | | <0.001 | <0.001 | 0.519 | 0.604 | -0.00001-0.00002 | <0.001 | <0.001 | 0.91 | 0.364 | -0.000009-0.00003 |
| Standard vs. minimalist | | <-0.001 | <0.001 | -6.56 | <0.001 | -0.00008- -0.00004 | <-0.001 | <0.001 | -6.84 | <0.001 | -0.00008- -0.00004 |
| Anticipation (No vs. Yes) | | <-0.001 | <0.001 | -0.36 | 0.717 | -0.00002-0.00001 | <0.001 | <0.001 | 0.447 | 0.655 | -0.00001-0.00002 |
| Approach Velocity (Covariate) | |  |  |  |  |  | <0.001 | <0.001 | 2.38 | 0.017 | 0.000004-0.00004 |
| Interaction barefoot/minimalist x Anticipation | | <-0.001 | <0.001 | -0.32 | 0.748 | ‚-0.00003-0.00002 | <-0.001 | <0.001 | -0.29 | 0.772 | ‚-0.00003-0.00002 |
| Interaction standard/minimalist x Anticipation | | <0.001 | <0.001 | 1.96 | 0.050 | 0.00000007-0.00005 | <0.001 | <0.001 | 2.03 | 0.043 | 0.0000009-0.00005 |
| **Muscle activity of gastrocnemius medialis at initial ground contact [uV]** | |  |  |  |  |  |  |  |  |  |  |
| (Intercept) | | 0.0002 | 0.0001 | 13.97 | <0.001 | 0.0002-0.0002 | 0.00004 | <0.001 | 1.24 | 0.217 | -0.00002-0.0001 |
| Barefoot vs. minimalist | | 0.00003 | <0.001 | 2.96 | 0.003 | 0.000009-0.00004 | 0.00003 | <0.001 | 3.85 | <0.001 | 0.00002-0.00005 |
| Standard vs. minimalist | | -0.00006 | <0.001 | -6.85 | <0.001 | -0.00008- -0.00004 | -0.00007 | <0.001 | -7.61 | <0.001 | -0.00008- -0.00005 |
| Anticipation (No vs. Yes) | | <-0.001 | <0.001 | -0.32 | 0.746 | -0.00002-0.00001 | 0.00001 | <0.001 | 1.49 | 0.135 | -0.000004-0.00003 |
| Approach Velocity (Covariate) | |  |  |  |  |  | 0.00005 | <0.001 | 5.45 | <0.001 | 0.00003-0.00007 |
| Interaction barefoot/minimalist x Anticipation | | <-0.001 | <0.001 | -1.39 | 0.166 | ‚-0.00004-0.000007 | <-0.001 | <0.001 | -1.33 | 0.185 | ‚-0.00004-0.000008 |
| Interaction standard/minimalist x Anticipation | | <0.001 | <0.001 | 1.33 | 0.183 | ‚-0.000008-0.00004 | <0.001 | <0.001 | 1.48 | 0.139 | ‚-0.000006-0.00004 |
| **Muscle activity of tibialis anterior at initial ground contact [uV]^e^** | |  |  |  |  |  |  |  |  |  |  |
| (Intercept) | | 0.0001 | <0.001 | 13.60 | <0.001 | 0.0001-0.0002 | -0.00003 | 0.00002 | -1.25 | 0.212 | -0.00008-0.00002 |
| Barefoot vs. minimalist | | <0.001 | <0.001 | 0.73 | 0.464 | -0.000008-0.00002 | 0.00001 | <0.001 | 1.92 | 0.055 | -0.0000003-0.00003 |
| Standard vs. minimalist | | 0.00004 | <0.001 | 5.81 | <0.001 | 0.00003-0.00005 | 0.00003 | <0.001 | 4.81 | <0.001 | 0.00002-0.00005 |
| Anticipation (No vs. Yes) | | -0.00001 | <0.001 | -2.10 | 0.036 | -0.00003- -0.0000009 | <0.001 | <0.001 | 0.37 | 0.710 | -0.00001-0.00002 |
| Approach Velocity (Covariate) | |  |  |  |  |  | 0.00005 | <0.001 | 7.26 | <0.001 | 0.00004-0.00007 |
| Interaction barefoot/minimalist x Anticipation | | <0.001 | <0.001 | 0.20 | 0.838 | ‚-0.00002-0.00002 | <0.001 | <0.001 | 0.31 | 0.761 | ‚-0.00002-0.00002 |
| Interaction standard/minimalist x Anticipation | | <-0.001 | <0.001 | -0.05 | 0.961 | ‚-0.00002-0.00002 | <0.001 | <0.001 | 0.14 | 0.893 | ‚-0.00002-0.00002 |

**Reference:**

79. E. A. Schmida, C. M. Wille, M. R. Stiffler-Joachim, S. A. Kliethermes, and B. C. Heiderscheit, “Vertical Loading Rate Is Not Associated With Running Injury, Regardless of Calculation Method.“ *Medicine & Science in Sports & Exercise* 54, no. 8 (2022): 1382-1388, http:// doi.org/10.1249/MSS.0000000000002917
